# Revisiting T7 RNA polymerase transcription *in vitro* with the Broccoli RNA aptamer as a simplified real-time fluorescent reporter

**DOI:** 10.1074/jbc.RA120.014553

**Published:** 2020-12-16

**Authors:** Zachary J. Kartje, Helen I. Janis, Shaoni Mukhopadhyay, Keith T. Gagnon

**Affiliations:** 1 Department of Chemistry and Biochemistry, Southern Illinois University, Carbondale, Illinois, USA; 2 Department of Biochemistry and Molecular Biology, Southern Illinois University School of Medicine, Carbondale, Illinois, USA

**Keywords:** RNA polymerase, T7, Broccoli, qPCR, RNA aptamer, transcription, fluorescence, rapid, BSA, bovine serum albumin, DMSO, dimethyl sulfoxide, PPase, pyrophosphatase, qPCR, quantitative PCR, RNAP, RNA polymerase, rNTP, ribonucleoside triphosphates, sgRNA, single-guide RNA

## Abstract

Methods for rapid and high-throughput screening of transcription *in vitro* to examine reaction conditions, enzyme mutants, promoter variants, and small molecule modulators can be extremely valuable tools. However, these techniques may be difficult to establish or inaccessible to many researchers. To develop a straightforward and cost-effective platform for assessing transcription *in vitro*, we used the “Broccoli” RNA aptamer as a direct, real-time fluorescent transcript readout. To demonstrate the utility of our approach, we screened the effect of common reaction conditions and components on bacteriophage T7 RNA polymerase (RNAP) activity using a common quantitative PCR instrument for fluorescence detection. Several essential conditions for *in vitro* transcription by T7 RNAP were confirmed with this assay, including the importance of enzyme and substrate concentrations, covariation of magnesium and nucleoside triphosphates, and the effects of several typical additives. When we used this method to assess all possible point mutants of a canonical T7 RNAP promoter, our results coincided well with previous reports. This approach should translate well to a broad variety of bacteriophage *in vitro* transcription systems and provides a platform for developing fluorescence-based readouts of more complex transcription systems *in vitro*.

Early methods to monitor transcription *in vitro* largely relied on the incorporation of radioactive nucleotides or detection of transcripts by hybridization-based methods (1, 2, 3, 4). These approaches continue to serve critical roles (5, 6, 7, 8). However, they have evolved to incorporate robotic handling, next-generation sequencing, fluorescence, and other multiplex readouts that have proven valuable for understanding biological mechanisms and screening for drugs (9, 10, 11, 12, 13, 14). Although these methods are powerful, they often involve specialized equipment or expertise that may not be readily accessible in a typical laboratory setting. Implementation of a straightforward screening approach that uses common laboratory equipment would enable cost-effective screening of focused small chemical libraries. Inspired by this need, we designed a simple, rapid throughput assay for monitoring transcriptional output in real-time with a fluorescent RNA aptamer reporter transcript. To test and develop this method as a potentially useful platform, we chose to characterize *in vitro* T7 RNA polymerase (RNAP) transcription, a common laboratory enzyme with well-studied promoter preferences and established reaction conditions.

For nearly half a century the mechanisms and applications of minimal viral RNA polymerases, such as those from the lambda, T4, T7, SP6, and SP8 bacteriophages, have served as a paradigm for transcription and RNA research (2, 15, 16, 17, 18, 19, 20, 21). Early experiments used bacteriophage transcription to help unravel the basic mechanisms of transcription and virulence (2, 15, 16, 17). These systems were subsequently reduced to their simplest components and co-opted for synthesis of RNA in the laboratory for biochemistry, molecular biology, and structural biology investigations (17, 18, 19, 22, 23, 24). The bacteriophage RNA polymerases, especially T7, have proven to be reliable work horses that continue to offer new insights into transcription as well as generate large quantities of RNA for research. The further optimization by affordable and high-throughput methods might be justified by the growing demand for pure and maximal transcript synthesis by modern molecular biology, structural biology applications, and even future mRNA therapeutics (21, 24, 25). Thus, T7 RNAP is an appropriate system to revisit and use as a benchmark for a rapid fluorescence-based screening method.

For *in vitro* T7 transcription reactions, the T7 RNAP is purified and combined with a DNA template that contains its cognate promoter and a downstream sequence encoding the RNA to be synthesized. A common consensus promoter is TAATACGACTCACTATA followed by one to three guanine nucleotides before the desired sequence for synthesis (19). In this study, we inserted the sequence encoding the Broccoli RNA aptamer (26, 27) to allow monitoring of RNA synthesis in real time by fluorescence detection. Upon proper folding and binding to the small molecule DFHBI-1T (3,5-difluoro-4-hydroxy-benzylidene imidazolinone), the short 49-nt Broccoli RNA aptamer fluoresces green with similar excitation and emission peaks as green fluorescent protein. This approach is similar to that taken by Hofer and colleagues previously, who fused the Spinach aptamer separated by a self-cleaving ribozyme to transcripts of interest (28). However, our focus was on low-cost synthetic DNA templates, high reproducibility, and robust fluorescence readout to characterize reaction conditions, polymerase properties, or promoter sequence. This led us to utilize only the short Broccoli RNA aptamer sequence itself in a hybrid template that is single stranded but possesses a double-stranded promoter (19). The Broccoli RNA aptamer has a very low dependence on magnesium and an increased thermostability compared with similar aptamers like Spinach (26, 28). The Broccoli RNA forms a G-quadruplex that relies on potassium or sodium ions to help stabilize structure and DFHBI-1T binding (29) (Fig. 1*A*). As a result of the RNA folded structure, the brightness and stability of Broccoli RNA fluorescence is also partially dependent on temperature, as will be the case for all folded aptamers (26, 29).

**Figure 1:**
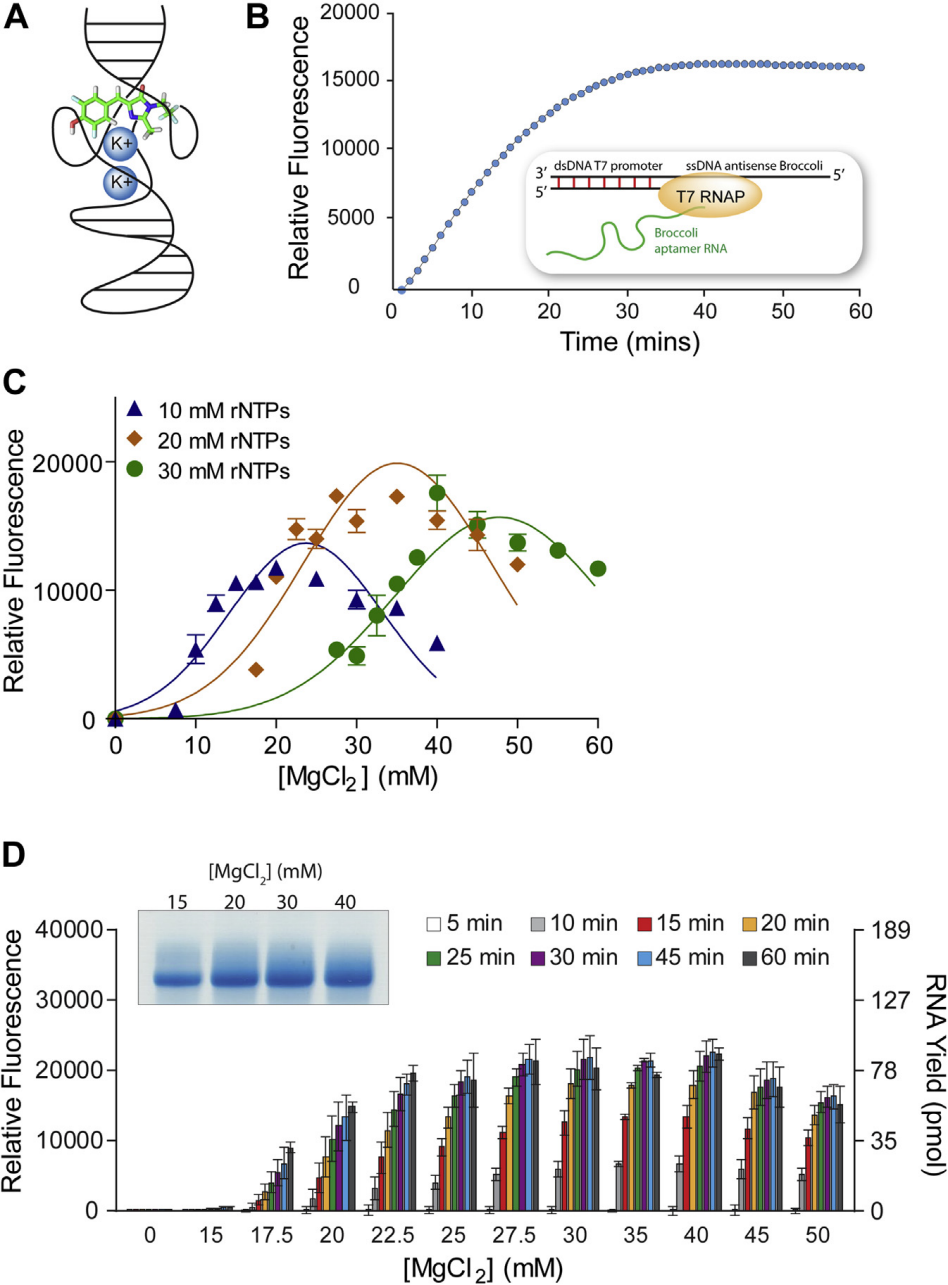
**Design of a fluorescent Broccoli RNA aptamer-based *in vitro* T7 transcription assay and the effect of covarying NTP and MgCl**_**2**_**concentrations.***A*, stylized illustration of Broccoli RNA aptamer G-quadruplex folding and DFHBI-1T binding. *B*, example data of fluorescence readout using T7 RNA polymerase and a hybrid ssDNA Broccoli RNA aptamer DNA template with a double-stranded T7 promoter. *C*, effect on relative fluorescence readout of T7 transcription upon titrating MgCl_2_ into reactions containing 10, 20, or 30 mM ribonucleoside triphosphates. The reaction fluorescence was taken at 15 min. Data were fit to a Gaussian distribution to better highlight trends in activity. R-squared values for fits were 0.82, 0.81, and 0.85 for 10, 20, and 30 mM MgCl_2_, respectively. Error bars are standard error of the mean (SEM). *D*, titration of MgCl_2_ into reactions containing 20 mM ribonucleoside triphosphates and relative fluorescence or RNA yield measured over time. The *inset* shows replicate samples pooled from 60-min fluorescent *in vitro* transcription reactions resolved by denaturing PAGE. Error bars are SEM.

Using 96-well plate screening, we confirmed the core buffer components necessary for efficient *in vitro* T7 transcription and explored the effect of several typical additives on transcriptional output. Fluorescence readout was correlated with denaturing polyacrylamide gel electrophoresis (PAGE) of selected samples, transcription of two additional longer RNAs, and comparison across several common buffer conditions. We also performed saturation mutagenesis of the T7 RNAP promoter with this method and found that our results generally coincided well with previous mutagenesis studies. The results of this study provide a resource to researchers interested in the properties of T7 RNAP or that use T7 RNAP for routine synthesis of RNA in the laboratory. The Broccoli RNA aptamer did possess some shortcomings and may have introduced some sequence-dependent biases in template transcription. However, other aptamers with distinct fluorescent properties and folding are now available that may address some of Broccoli’s weaknesses, including Mango, Pepper, and o-Coral (30, 31, 32). The ability of this method to recapitulate and confirm many of the commonly known properties of *in vitro* T7 RNAP transcription suggests that this approach should translate well to a variety of other bacteriophage RNA polymerases. It should also serve as an initial platform for developing screens for more complex eukaryotic *in vitro* transcription systems.

## Results and discussion

### An *in vitro* transcription assay with rapid fluorescent readout

To enable cost-effective, rapid, and direct readout of transcription, we selected the Broccoli RNA aptamer for its compact size, relative thermal stability, low magnesium dependence, and bright green fluorescence. Our design is similar to that reported previously by Jäschke and co-workers (28). However, we focused on a generalizable assay amenable to most laboratories without the potential shortcomings of the Spinach RNA aptamer or complexity of ribozyme modules. We used a simple hybrid ssDNA template structure comprising a single-stranded antisense sequence encoding Broccoli RNA with only the T7 RNAP promoter region being double stranded. This template structure was originally described by the Uhlenbeck laboratory and is widely used for efficient transcription of small RNAs from synthetic DNA oligonucleotides (19). Upon initiation of the reaction in the presence of DFHBI-1T, green fluorescence is observed within the first few minutes. Fluorescence was typically measured every minute in a standard quantitative PCR (qPCR) instrument in small 10-μl reactions in a 96-well format.

By this method, Broccoli RNA aptamer synthesis and fluorescence are observed in real time (Fig. 1*B*), which provides a relative measurement of total RNA production over time. In most experiments, RNA transcripts from fluorescent reaction replicates were pooled and resolved by denaturing PAGE to compare staining on a gel to fluorescence-based detection. Resolution of RNA transcripts by denaturing PAGE sometimes resulted in multiple bands or higher-molecular-weight smearing. It is possible that the strong quadruplex nature of the Broccoli RNA aptamer (29) resulted in varying degrees of denaturation across experiments. Based on standard curves, the concentration of RNA produced can be estimated by the relative amount of fluorescence observed or the intensity of band staining by PAGE (Fig. S1, *A*–*B*).

### Codependence of MgCl_2_ and ribonucleoside triphosphate concentrations by T7 RNAP

The overall concentration of MgCl_2_ and ribonucleoside triphosphates (rNTPs), as well as their concentrations relative to one another, has been known to impact *in vitro* T7 RNAP transcription efficiency (22, 33). The T7 RNAP enzyme itself requires a certain concentration of free magnesium to function, and the triphosphate groups on rNTPs can stoichiometrically chelate magnesium ions. It has been suggested that MgCl_2_ should be 6 mM above the concentration of rNTPs for optimal efficiency (22, 33). Thus, for standard transcription reactions the concentration of each rNTP is usually set at 4 mM (16 mM total rNTPs) while MgCl_2_ is set at a final concentration of 20 mM (19). We decided to revisit this fundamental aspect of *in vitro* T7 transcription. We varied the MgCl_2_ concentration widely across total rNTP concentrations of 10, 20, and 30 mM under otherwise standard conditions (Fig. 1*C*). Maximal RNA synthesis at each rNTP concentration appeared to be centered on a MgCl_2_ concentration that was approximately 10 mM higher than the total rNTP concentration. This was observed at all three rNTP concentrations, suggesting that aptamer reporter dependence on Mg^2+^ does not contribute significantly to this effect. Nonetheless, although the Broccoli RNA aptamer was demonstrated to only require very low millimolar concentrations of Mg^2+^ (26, 29), its fluorescence may be influenced by increasing Mg^2+^ in our reactions and therefore may not translate to typical T7 RNAP transcription reactions.

Although 30 mM rNTPs should support greater RNA synthesis, the conditions that produced the most consistent yield appeared to be 20 mM rNTPs with 30 mM MgCl_2_ (Fig. 1*D*, Fig. S2). These results suggest that both limiting and excessive MgCl_2_ can inhibit the activity of T7 RNAP. To confirm this, we instead held MgCl_2_ constant at 20 mM and titrated rNTPs from 10 mM up to 35 mM (Fig. S3*A*). The RNA yield was severely reduced when rNTP concentrations exceeded MgCl_2_ concentration. In addition, 50 mM MgCl_2_ was inhibitory in transcription reactions with 20 mM rNTPs when transcribing 7SK-Broccoli, a ∼450-nt template that encodes the human 7SK RNA sequence fused to the Broccoli RNA aptamer (Fig. S4*A*). Precipitates of Mg^2+^ and pyrophosphate are common during *in vitro* transcription reactions and can potentially alter the effective free Mg^2+^ concentration available for T7 RNAP. However, precipitation might be neutral or beneficial since the reduction of free Mg^2+^ would conceivably be proportional to consumption of rNTPs and would also remove potentially inhibitory pyrophosphate from solution. We included inorganic pyrophosphatase (PPase) in fluorescence reactions to prevent cloudiness and maintain accurate reading. Therefore, we investigated the effect of PPase at three different rNTP concentrations. When transcription products were resolved by denaturing PAGE, we did not observe any increase in transcription yield by the addition of PPase (Fig. S3*B*). Thus, we concluded that PPase or the formation of magnesium pyrophosphate precipitates is unlikely to alter the interpretation of results or cause unforeseen issues in fluorescence-based transcription screens. Together, these results support the importance of balancing and properly covarying rNTP and MgCl_2_ concentrations. These results are similar to previous reports but suggest that a slightly elevated concentration of Mg^2+^ during *in vitro* T7 transcription reactions could be beneficial.

### DNA template and T7 RNAP concentration effects

**Figure 2:**
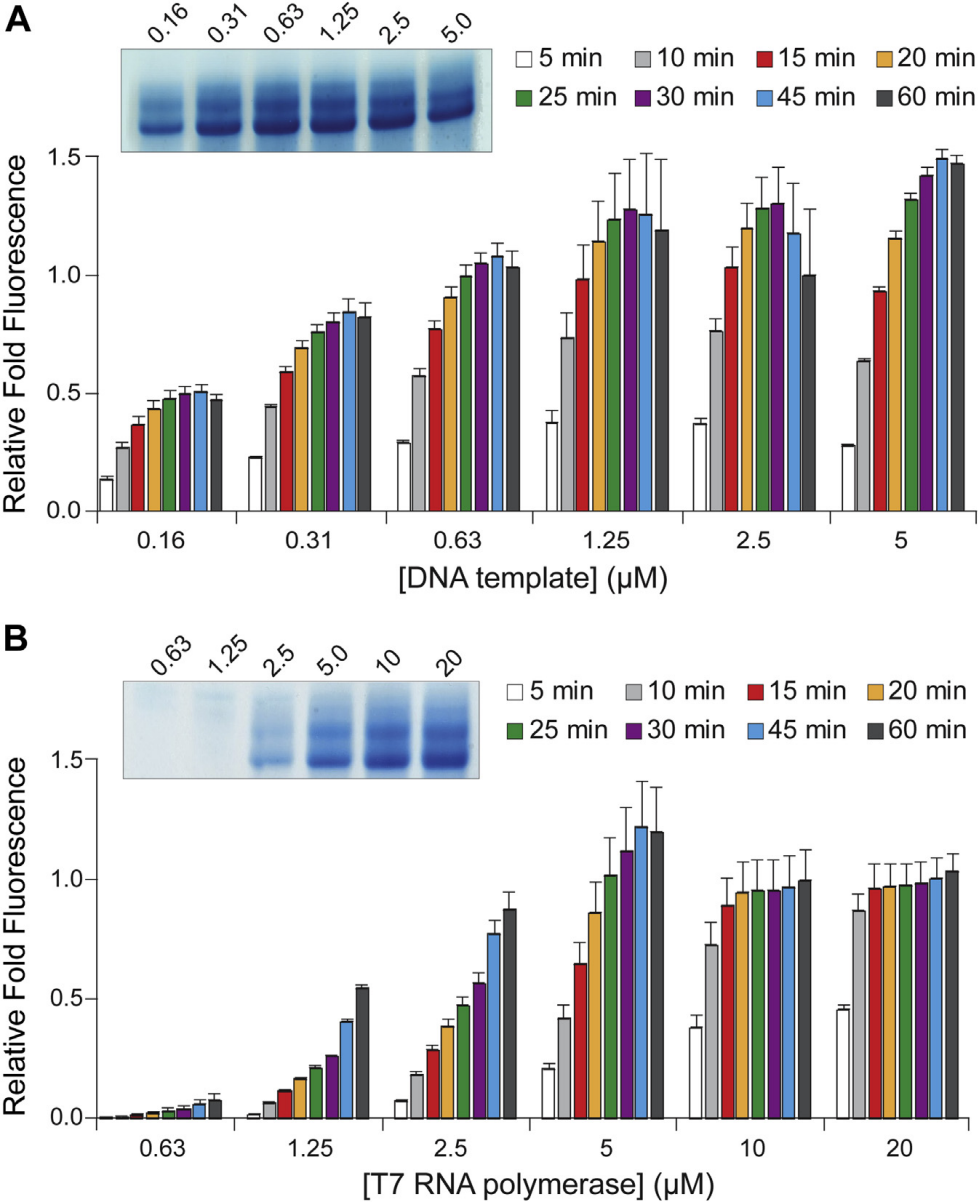
**Effect of DNA template and T7 RNAP concentration on *in vitro* T7 transcription efficiency.***A*, increasing concentrations of hybrid ssDNA Broccoli template in fluorescent T7 transcription reactions. *B*, increasing concentrations of T7 RNAP in fluorescent T7 transcription reactions. For all panels, relative fold fluorescence is normalized to standard conditions. *Insets* show three pooled reactions from 60 min time points resolved by denaturing PAGE. Error bars are SEM.

We decided to test other conditions fundamental to *in vitro* T7 RNAP transcription, which included the concentration of DNA template, T7 RNAP, monovalent ions, and polycations, as well as temperature and pH. Beginning with our standard conditions, we titrated the ssDNA hybrid template for Broccoli RNA transcription. As expected, the amount of DNA template proportionally increased the overall production of RNA transcript when the template was limiting (Fig. 2*A*). Transcription plateaued around 1.25 μM of template. Of importance, the amount of RNA made at each individual concentration of template also plateaued, indicating that longer incubations will not necessarily result in greater amounts of RNA when the template is limiting. Conversely, excess template did not seem to inhibit the reaction. When transcribing 7SK-Broccoli from a linearized plasmid template we observed a similar proportional increase in transcriptional output up to the final amount of 2 μg (0.2 μg/μl) at which point the total output appeared to begin plateauing (Fig. S4*B*). This is similar to the concentration of 1 to 3 μg of linearized plasmid per 20 μl reaction that is recommended in common commercial T7 transcription kits, such as the Ampliscribe T7-*Flash* Transcription Kit (Epicentre/Lucigen). In contrast to the synthetic DNA template encoding Broccoli, transcription of the longer 7SK-Broccoli from a linearized plasmid increased steadily over time at low template concentrations until reaching 1 μg of template (0.1 μg/μl) (Fig. S4*B*), suggesting that longer incubations may be required to produce more RNA product when plasmid DNA templates are limiting or when longer RNAs are transcribed.

The amount of T7 RNAP affected not only the amount of RNA produced but also the rate at which it was produced (Fig. 2*B*). The transcription reaction reached completion much sooner when higher concentrations of T7 RNAP were present. For example, the reaction appears to have produced its maximum amount of RNA product at 30 min with 5 μM T7 RNAP while the product continues to increase steadily up to 60 min with 2.5 μM T7 RNAP. We also noted that Broccoli fluorescence was quenched at the highest concentrations of T7 RNAP, even though increasing amounts of transcript appeared to be synthesized when visualized by denaturing PAGE. Since Broccoli RNA aptamer fluorescence is sensitive to salts and DFHBI-1T interaction, it is possible that elevated concentrations of protein can partially titrate these or other components in the reaction. Overall, greater amounts of enzyme appear to proportionally increase transcript production independent of other factors. Nonetheless, exceeding 10 μM T7 RNAP under our standard conditions did not produce substantially greater yields and excess RNA polymerase has been reported to inhibit large-scale transcriptions (22). Thus, suboptimal concentrations of either template or T7 RNAP will limit overall synthesis of RNA, although *in vitro* transcription efficiency appears much more sensitive to T7 RNAP concentrations.

### Temperature and pH dependence of T7 RNAP

**Figure 3:**
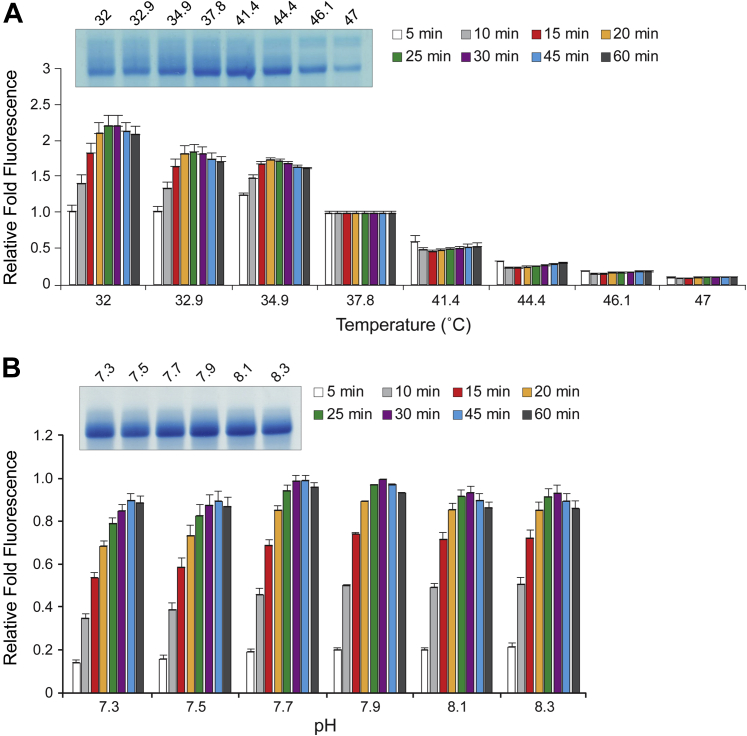
**Effect of temperature and pH on *in vitro* T7 transcription efficiency.***A*, T7 transcription efficiency at a range of temperatures. Fluorescence at each time point was normalized to standard conditions and 37.8 °C. *B*, T7 transcription efficiency at a range of pH, with 7.9 being the standard reference condition. For all panels, relative fold fluorescence is normalized to standard conditions. *Insets* show three pooled reactions from 60 min time points resolved by denaturing PAGE. Error bars are SEM.

When we tested transcription as a function of temperature using a temperature gradient setting on our qPCR instrument, we observed reduced fluorescence with increasing temperature (Fig. 3*A*). This was not surprising given that Broccoli RNA, like other folded RNA structures, is more stable at lower temperatures and therefore is expected to bind DFHBI-1T more stably to produce greater fluorescence. When pooled reactions were resolved by denaturing PAGE, transcript production was greatest around the 37.8 and 41.4 °C temperature points. Thus, it appears that transcription is most efficient at slightly elevated temperatures. Although the fluorescence-based melting temperature (*T*_m_) of Broccoli was determined to be ∼47 °C previously and it was shown to be ∼90% folded at 37 °C (29), thermal dependence is a potential shortcoming of RNA folded structures as reporter molecules.

A critical aspect of activity for many enzymes is proton concentration. We tested a small pH range of one unit, from 7.3 to 8.3, which represents an order of magnitude change in proton concentration. (Fig. 3*B*). The efficiency of the reaction was not dramatically impacted within this pH range but appeared slightly higher around pH 7.9, which is our standard condition and very similar to the optimal pH of 8.1 reported previously (19). Denaturing PAGE analysis supported these results. It is notable that DFHBI-1T’s photophysical properties are altered below pH 7.0 (27), which would likely limit its ability to be used as a reporter system at a lower pH.

### Sodium chloride and spermidine in T7 RNAP reactions

**Figure 4:**
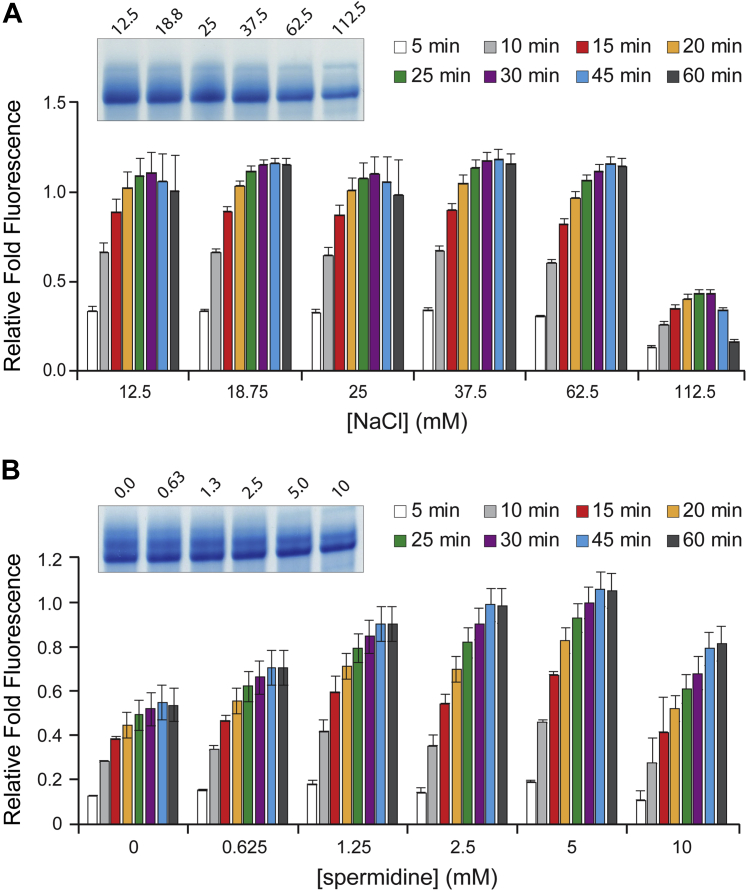
**Effect of NaCl and spermidine concentrations on *in vitro* T7 transcription efficiency.***A*, increasing concentrations of NaCl in standard fluorescent T7 transcription reactions. *B*, increasing concentrations of spermidine in standard fluorescent T7 transcription reactions. For all panels, relative fold fluorescence is normalized to standard conditions. Insets show three pooled reactions from 60 min time points resolved by denaturing PAGE. Error bars are SEM.

Previous studies have observed that monovalent salt ions, such as sodium and chloride, can have a strong inhibitory effect on T7 RNAP activity (19). Chloride ions have been reported to compete with the DNA template for anion-binding sites on the polymerase, increasing the Michaelis constant (*K*_M_) between the polymerase and the promoter (34). Conversely, spermidine and related polycations have been reported to improve T7 transcription reaction yields (35). We decided to screen NaCl concentrations starting from 12.5 up to 112.5 mM (Fig. 4*A*). These reactions also contained 1 mM KCl to minimally stabilize the Broccoli RNA G-quadruplex structure (29). The fluorescence signal was largely unchanged until the highest concentration was reached. However, RNA production by denaturing PAGE revealed a moderate but steady decrease in yield as NaCl concentrations increased. This discrepancy is likely explained by the monovalent salt dependence of the Broccoli RNA aptamer, which possesses a G-quadruplex core that is stabilized by both sodium and potassium ions. Although potassium ions are preferred for G-quadruplex coordination, sodium ions can substitute at higher concentrations (29). In addition, small local variations in the quadruplex core structure can have significant effects on fluorescence owing to DFHBI-1T stacking onto the quadruplex (36). Although increasing salt concentrations can globally improve RNA structure and stability, the high concentration of MgCl_2_ in our reactions likely provides strong neutralization of backbone repulsion and results in a stably folded Broccoli RNA aptamer. Thus, steady decreases in synthesis were likely countered by proportional increases in the stability or folding of the quadruplex core itself and, therefore, increased fluorescence of the aptamer. For this reason, we did not test the effect of KCl since it has an even more pronounced effect on Broccoli G-quadruplex stability (29). In summary, lower concentrations of NaCl were conducive to higher yields and fluorescence-based measurements using our method were inaccurate when comparing conditions with differential NaCl concentrations. The use of aptamers that do not possess G-quadruplex structure, such as o-Coral and Pepper (31, 32), may provide a solution for reducing monovalent salt dependence in screening assays.

Spermidine is usually considered a critical component for *in vitro* transcription reactions (19, 35). Spermidine is a polyamine that potentially interacts with inhibitory polyanions and has been shown to enable the polymerase to dissociate from the DNA template and initiate new DNA chain synthesis (35). In addition, spermidine has been reported to prevent the inhibition of RNA synthesis by exogenous RNA (37) and to improve the overall efficiency of T7 RNAP transcription *in vitro* (19). In our assay, a moderate amount of spermidine, around 2 to 5 mM, appeared to improve overall RNA yield but became inhibitory at 10 mM (35) (Fig. 4*B*). The benefits of polyamines like spermidine in transcriptional proficiency have already been established, especially for small, structured transcripts similar to Broccoli RNA. The concentrations of Mg^2+^ ions and spermidine could offer synergistic effects and may be codependent upon each other, although this was not explored here.

### Dithiothreitol, dimethyl sulfoxide, nonionic detergent, and molecular crowding in T7 RNAP reactions

Our initial screens coincided relatively well with previously established conditions for *in vitro* T7 RNAP transcriptions. Thus, we decided to further investigate several other conditions relevant to our assay and additives often cited to be beneficial to *in vitro* transcription. Dithiothreitol (DTT) is a reducing agent that protects against protein oxidation and is routinely included in T7 RNAP preparations and *in vitro* transcription reactions. Early viral RNA polymerase reactions included up to 10 mM 2-mercaptoethanol or 5 mM DTT (17, 22). Anecdotally, it is often recommended that 1 to 2 mM fresh DTT be added to frozen T7 RNAP stocks before use, or every few months. We found that additional DTT did not substantially impact transcription at up to 10 mM, suggesting that its addition at higher concentrations is unlikely to be necessary for robust transcription when using fresh stocks of T7 RNAP (Fig. S5*A*).

The small molecule DFHBI-1T, which is responsible for Broccoli fluorescence, is most soluble in dimethyl sulfoxide (DMSO). Therefore, relatively low concentrations of DMSO were present in our transcription reactions when monitoring fluorescence. This solvent appeared to be helpful in modest amounts (>1.23%), whereas inhibitory at higher concentrations (Fig. S5*B*). This could be due to improved solubility of DFHBI-1T. However, gel electrophoresis did not show improvements in transcription at DMSO levels up to a final concentration of 10.6%. Of interest, the addition of up to 15% DMSO was previously reported to increase the efficiency of T7 transcription through altered protein structure (38). These results suggest that the presence of DMSO may not be faithfully investigated in our assay given the likelihood that fluorescence is a function of DFHBI-1T solubility. Thus, the solubility of aptamer–fluorophore combinations may be an important consideration when developing fluorescence-based aptamer readout for transcription assays.

Nonionic detergents like Triton X-100 have been considered beneficial for successful transcription (17, 19, 35, 39). Triton X-100 was reported to improve RNA production in transcription systems (35, 40) as well as increase initiation kinetics in specific conditions (34). In our screen using the Broccoli RNA aptamer, Triton X-100 did not seem to affect *in vitro* T7 transcription yield, either by fluorescence-based measurements or PAGE (Fig. S6*A*). This result agrees with a previous study (34) but differs from another that saw an improvement in yield of up to 50% (39). Triton X-100 is also added to reduce adsorption to plastic reaction vessels and its benefit may depend on the reaction scale.

Polyethylene glycol (PEG) and bovine serum albumin (BSA) are common additives that have both been associated with successful *in vitro* transcription protocols for decades (35, 40, 41). They are expected to behave like crowding agents that can increase the effective concentration of reaction components to improve intermolecular interactions. PEG has been reported to improve transcriptional yields up to 50% for large-scale transcriptions (19, 22). In contrast, lower-molecular-weight PEG_200_ has recently been reported to reduce T7 RNAP transcription (42). PEG_8000_ appeared to have little or no effect on *in vitro* T7 transcription under our standard conditions (Fig. S6*B*). BSA seemed to offer some improvements in transcription kinetics and yield by fluorescence measurements but this was not obvious by gel electrophoresis (Fig. S6*C*). It is possible that these components would give a more observable effect at reduced concentrations of enzyme or substrate. Likewise, in our standard conditions both PEG (2%) and BSA (50 μg/ml) are normally present. Since they may have a redundant effect, removing only one may not significantly impact the overall transcription.

In our assay, we did not expect these agents to substantially affect folding of the Broccoli RNA aptamer itself since it is a compact, intramolecularly folded structure and PEG_8000_ and BSA are rather large molecules (29, 36). Nonetheless, molecular crowders like PEG_1000_ and PEG_8000_ have been reported to enhance the intramolecular folding and activity of certain moderate to large RNAs and ribozymes, respectively, by the excluded volume effect (43, 44, 45). In our assay, the correlation between fluorescence and transcription is therefore unclear. However, PAGE results support the conclusion that there were no substantial changes in transcriptional efficiency (Fig. S6).

Of interest, crowding agents like PEG may also be codependent on other reaction components in our assay, such as salts. For example, increased sodium chloride concentrations might destabilize protein–nucleic acid interactions but increase Broccoli RNA aptamer folding (45). The concentration of certain components like PEG, NaCl, or MgCl_2_ might be valuable for studies focused on enzyme mechanisms, such as T7 RNAP interactions with the promoter (46, 47). In addition, these crowding agents can have other properties beneficial to reactions. For example, BSA may help reduce adhesion of enzymes like T7 RNAP to plastic surfaces. Covarying PEG, BSA, or other crowding agents independently with other reaction components might provide a rationale for the apparent discrepancies between multiple reports. However, our assay may not be ideal for such an analysis owing to the potential effects of molecular crowding and ion concentrations on Broccoli aptamer folding and fluorescence.

### Comparing common *in vitro* T7 RNAP transcription conditions

**Figure 5:**
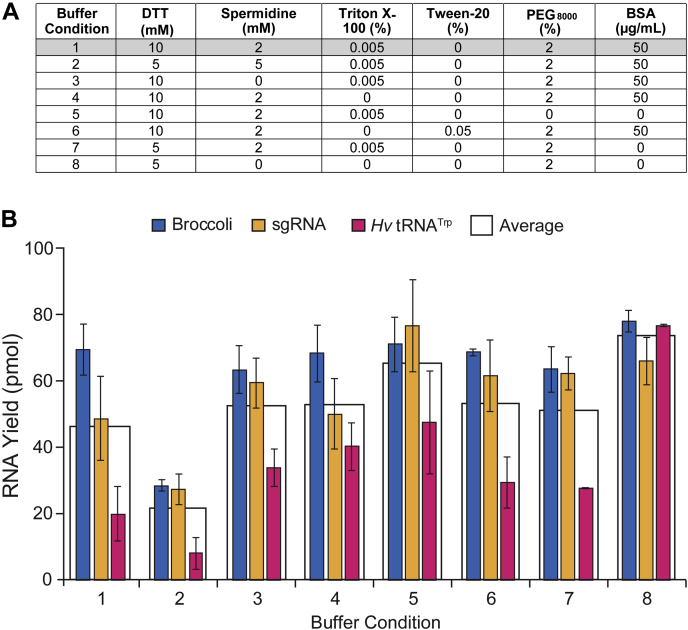
**The effect of varying common reaction additives on the efficiency of *in vitro* T7 transcription for different templates and RNA products.***A*, table of reaction additives commonly used and their concentration. Buffer Condition 1 represents standard reaction conditions. Other reaction components not shown are at the concentrations noted for standard conditions (see Methods). *B*, *in vitro* T7 transcription yield quantified by band size and intensities from denaturing PAGE. The yield was determined using a standard curve of Broccoli RNA aptamer after staining with methylene blue and is therefore based on Broccoli RNA concentrations (see Fig. S1). Error bars are SEM.

A practical outcome of testing the contribution of individual reaction conditions and buffer components is the identification of guidelines for optimal T7 transcription. To determine if our assay could provide more insight, we compared several reaction conditions, including published recipes, with varying additives using PAGE analysis. We transcribed three distinct RNAs from different templates using T7 RNAP to help determine if any trends might be generalizable. The RNAs transcribed were the Broccoli RNA aptamer, a CRISPR-Cas9 single-guide RNA (sgRNA) (48), and a tRNA^Trp^ from *Haloferax volcanii* (49). Broccoli is a short, highly structured 49-nt RNA aptamer, the sgRNA is a 99-nt-long moderately folded RNA with a poorly structured 20-nt 5' guide region, and tRNA^Trp^ is a highly structured 75-nt tRNA. These RNAs represent a small but diverse group that might typically be transcribed in the laboratory. Eight separate conditions were compared (Fig. 5*A*), and components not listed were held constant at concentrations noted for our standard reaction conditions. Thus, buffering agent (Tris-HCl), pH, DNA template, NaCl, MgCl_2_, DMSO, rNTPs, temperature (37 °C), and T7 RNAP were all held constant while the common additives DTT, spermidine, Triton X-100, Tween 20, PEG_8000_, and BSA were varied. Condition 1 represented our standard control reaction condition that was used to compare with other component variations.

By quantifying stained RNA bands on a denaturing PAGE gel and comparing with our standard curve (Fig. S1*B*), we calculated approximate transcription yields (Fig. 5*B*). Our standard condition (buffer 1) was excellent for Broccoli RNA production, but synthesis of sgRNA was clearly reduced and tRNA^Trp^ production was minimal. Buffer 2, which contained all additives except Tween 20, performed the worst. The overall best-performing condition was buffer 8, which gave consistently robust RNA production for all three templates, calculated to be approximately equivalent to 80 pmol of Broccoli RNA from a 20-μl reaction. Of interest, buffer 8 has no spermidine, Triton X-100, or Tween 20. Spermidine appeared to be beneficial in our individual component screens. It is notable that buffer 5 was absent of Tween 20, PEG, and BSA but gave the second highest RNA production overall.

Buffers 3, 4, 6, and 7 performed similarly, producing as much or more RNA as our standard buffer 1. Buffer 3 contained no spermidine while 4, 6, and 7 did; buffers 3 and 7 contained Triton X-100 while 4 and 6 did not; and buffers 3, 4, and 7 contained no Tween 20 while buffer 6 did. These data support the performance of buffer 8, which provides high RNA production in the absence of each of these components. We note that additives and buffer components can have unforeseen synergistic effects, potentially both positive and negative. Testing of more components, combinatorial conditions, and a more diverse set of templates with fluorescence-based and traditional readouts could further optimize transcription conditions and could be accomplished by fusing the specific sequence of interest to Broccoli with a self-cleaving ribozyme linker, similar to that of Jäschke and co-workers (28). Nonetheless, our limited comparative analysis surprisingly suggests that minimal buffer compositions are likely to work well with broad types of RNA transcripts and templates. Although some of our fluorescence-based results predicted that certain additives would not be beneficial, such as Triton X-100 or BSA, they did not predict that lack of spermidine would be efficient. Together, these results also suggest that various aptamers should be tested in the context of other sequences in case this impacts folding, fluorescence, and interpretation of transcription data. Embedding the aptamer in an insulator structure, such as the F30 three-way junction scaffold (50), may help avoid folding and sequence-dependent biases in a manner similar to the use of a self-cleaving ribozyme (28).

### Fluorescence-based mutagenesis scanning of the T7 RNAP promoter sequence

**Figure 6:**
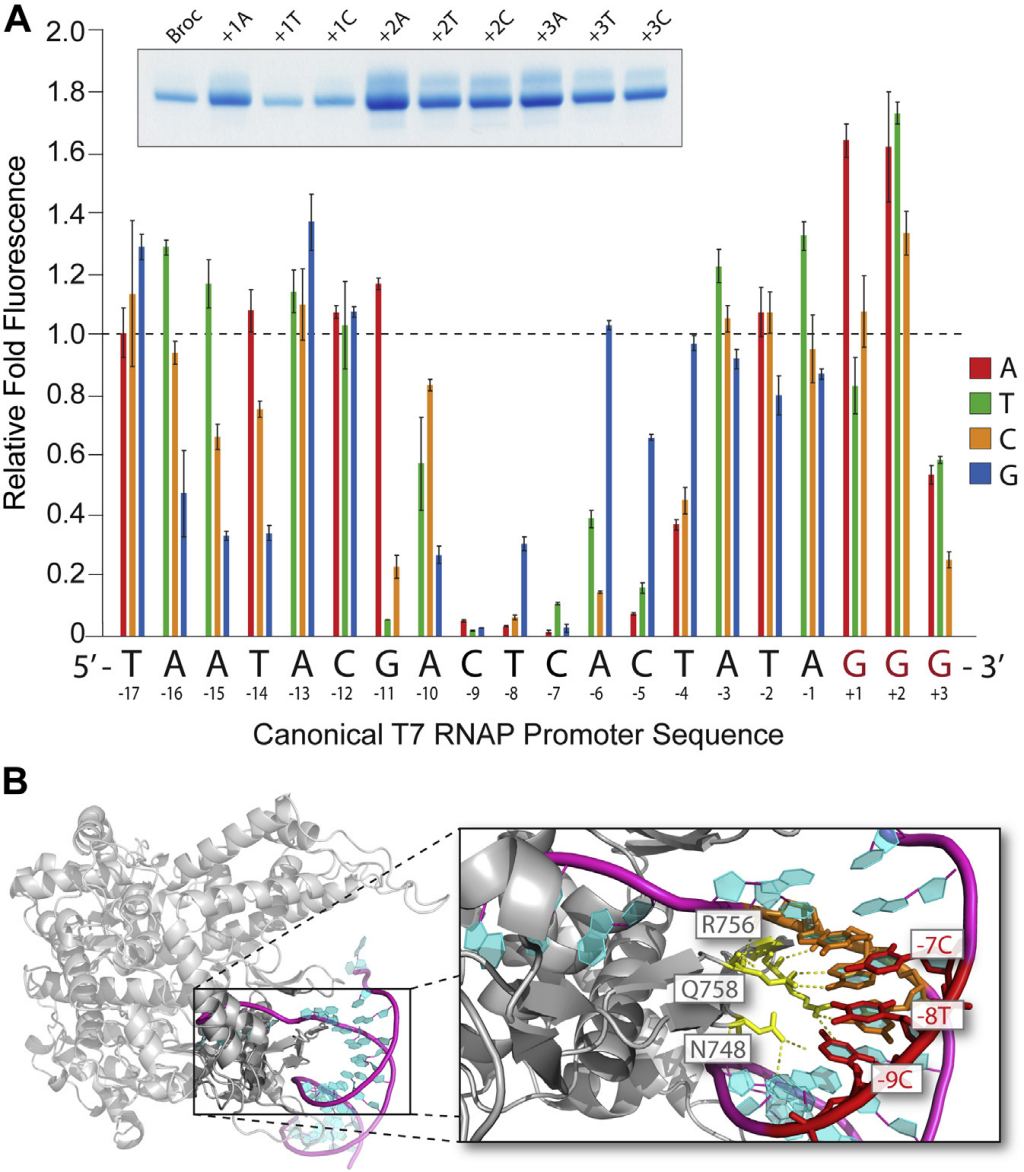
**Transcription efficiency of systematically mutated T7 promoter quantified by fluorescent Broccoli RNA synthesis.***A*, systematic mutation of the canonical T7 promoter by changing each nucleotide one at a time across the promoter sequence. At each nucleotide position in the sense strand of the promoter, the effect of substituting a different base pair is shown (the antisense promoter strand also contained a compensatory base change). The canonical core T7 promoter sequence with GGG from +1 to +3 is shown on the x-axis. Relative quantified fluorescence, and therefore transcription efficiency, is represented on the y-axis and normalized to the canonical promoter activity. Nucleotides in the promoter are numbered starting with the most 5’ nucleotide in the sense strand as position −17 to the most 3’ nucleotide as position +3. The inset shows three pooled reactions from 60 min time points resolved by denaturing PAGE for mutants at +1, +2, +3. Error bars are SEM. *B*, a close-up view of the T7 RNAP interaction with its canonical promoter sequence taken from a previously published crystal structure (Protein Data Bank: 1CEZ) (51). The importance of the central base pairs (−7, −8, and −9) to T7 RNAP recognition are highlighted and polar contacts to T7 RNAP or water molecules are shown. Nucleotides and key amino acids are indicated.

Real-time fluorescence readout might facilitate rapid screening of T7 RNAP enzyme and promoter DNA variants. To investigate the potential utility of our assay for rapid mutagenesis screening, we *in vitro* transcribed a set of 60 ssDNA hybrid templates that encoded Broccoli RNA and possessed mutant double-stranded promoters. Each mutant represented a single base-pair change to one of the three other base pairs, which is a mutagenesis method known as saturation mutagenesis (11). For example, the first three mutants contained an A, C, or G in place of T at the first nucleotide of the T7 promoter. Fully complementary promoter oligos were annealed to each mutant to create promoters with an uninterrupted double helix. This was performed at all 20 positions in the promoter starting at the 5' end at position −17 and ending at the 3' end at position +3 (Fig. 6*A*). The T7 RNAP promoter sequence in common use has been systematically mutagenized previously to identify sequence elements important for activity (3, 4, 11, 14). These previous studies represented a reliable test to benchmark our approach.

We observed that the identity of the first base pair in the promoter was not critical at position −17. However, positions −16 through −14 preferred to be an A-T or T-A base pair (where the first nucleotide is on the nontemplate, or sense, strand and the second nucleotide is on the template, or antisense, strand). Any base pair seemed to be well accommodated at positions −13 and −12. In contrast, position −11 strongly preferred a purine nucleotide on the nontemplate strand, as did position −6. Position −10 is usually an A-T base pair but could accommodate T-A or C-G pairs to some degree. Positions −9 to −7, representing C-G, T-A, and C-G base pairs in the center of the duplex, were essential for promoter activity and could not be substituted with any other nucleotides. The results described above for positions −15 to −6 all matched those of Diaz and co-workers (3) very closely, despite our use of a fluorescence reporter as opposed to traditional methods like gel electrophoresis and linearized plasmid templates. They also correlated well with the results of Patwardhan and co-workers, who used pooled promoter variants and next-generation sequencing quantification (11). For example, we also observed mutation of −16A and −15A to T with little effect, mutation of −13A to any other nucleotide with little consequence, mutation of −11G to A with no effect, and no tolerance for mutations at positions −9, −8, and −7 (11).

The invariant nucleotides at positions −9 to −7 can be rationalized by inspection of an existing crystal structure of T7 RNAP bound to its cognate DNA promoter (Fig. 6*B*). Specific recognition of the G, A, and G bases on the template strand at these base-pair positions is mediated primarily through hydrogen bonding with T7 RNAP amino acids Q758 and R756, with some help from N748 (46, 51). The importance of these central base pairs was also recognized by other previous studies (3, 11, 14, 46), thereby supporting the reliability of our assay. Position −5 preferred a G-C or C-G base pair and position −4 performed well with substitution of T for a G nucleotide, meaning a T-A or G-C base pair was equivalent, but not an A-T or C-G base pair. The remaining positions (−3 to −1) had no base-pair preference over the common promoter sequence.

Our assay results deviated from those of previous studies when comparing results at positions +1 to +3. Indeed, these positions instead seemed to show enhanced transcription up to almost 2-fold when mutated. Position −1 preferred a T-A base pair (over A-T), position +1 preferred an A-T base pair (over G-C), and position +2 performed very well with any other nucleotide other than the canonical G. Frame shifting from +1 to +2 could explain the high transcription efficiency when the +1 position is mutated to a C or T, as suggested by Imburgio and colleagues (4). However, it is unclear if this could account for high efficiency of other mutants. Of interest, substituting the G at position +3 for any other nucleotide resulted in an ∼50% or greater loss in fluorescence.

A potential explanation for differences in our promoter analysis at the +1 through +3 positions is the use of an ssDNA hybrid template as opposed to a dsDNA template. Thus, we repeated promoter mutagenesis at these positions using a dsDNA template. However, the results were very similar and consistent for both template formats (Fig. S7*A*) despite the observation that dsDNA templates mediated faster transcription kinetics (Fig. S8). Of interest, mutations at the +3 position had no impact on transcription yield by gel electrophoresis and instead appeared to increase yield, especially when G was replaced with A (Fig. S7*B*). We propose that dependence of G at position +3 for high fluorescence is linked to folding of the Broccoli aptamer since it is a G-quadruplex structure (36, 52).

Our results generally agreed quite well with Patwardhan and colleagues (11) but there were some differences when considering the +1 to +3 GGG nucleotides. Template sequence-specific effects might account for these differences in our study (19). For example, the Broccoli RNA aptamer is G-rich and begins with GAG at the 5' end. Nonetheless, the overall strong congruence between previous studies and our results suggests that our method should prove to be reliable and robust for translation to other *in vitro* transcription systems.

### Conclusions

We have presented a method using the Broccoli RNA aptamer as a direct and rapid fluorescent readout for *in vitro* transcription that should be straightforward to implement by other researchers for a variety of bacteriophage polymerases. We efficiently quantified how RNA synthesis by T7 RNAP *in vitro* is affected by reaction conditions, buffer components, and promoter sequence. As expected, components such as T7 RNAP and DNA template concentration, as well as rNTP and MgCl_2_ concentrations and ratios, greatly impacted product yield. Common additives, such as spermidine, Triton X-100, PEG, BSA, and DTT, had varying degrees of success in our assay. Of interest, the most reliable reaction conditions that we tested lacked many of these common additives. Thus, it is possible that a relatively simple buffer may be sufficient to support robust transcription for most templates. However, our fluorescence-based assay may not be ideal for complete characterization owing to the variability that can arise from different template sequences. Similarly, systematic promoter mutagenesis with our assay coincided well with most previous results, adding confidence to the reliability of our approach. However, they differed near the end of the promoter (+1 to +3) adjacent to the Broccoli RNA aptamer template sequence. Although our approach uses direct fluorescence-based readout and focuses on one transcription reaction per mutation, the reliance on aptamer reporter sequence, folding, and fluorescence likely contributed to some deviation from previous studies.

Potential caveats of our assay include the relatively short length of the Broccoli RNA aptamer, as well as specific structural features of RNAs like the Broccoli aptamer and tRNA. In addition, T7 RNAP transcription was typically initiated from an ssDNA hybrid template with only a double-stranded promoter. Thus, it is unclear if there will be a full generalization of the results obtained with our synthetic templates and short Broccoli RNA to much longer transcripts derived from linearized plasmids or fully double-stranded templates. The assay may also be limited to a range of conditions, such as pH, temperature, and ion concentrations. By embedding the aptamer sequence within a scaffold, or at the end of a target transcript and separated by insulators like self-cleaving ribozymes (28), some shortcomings may be mitigated (32, 50). Additional aptamer–fluorophore pairs that may be valuable as *in vitro* transcription reporters include Mango/TO1-biotin, Pepper/HBC, and o-Coral/Gemini-561. Mango, which binds a thiazole orange derivative, causes fluorescence in the green-yellow, orange, or red range, depending on the thiazole orange derivative bound (30, 53, 54). Pepper is a recently selected aptamer that binds various derivatives of HBC, molecules that borrow similar structural and fluorescence features to that of DFHBI-1T, which binds Broccoli (26, 32). Pepper can create fluorescence of HBC derivatives across a range of colors, from cyan to red. O-Coral is an aptamer that binds a dimeric and self-quenched derivative of sulforhodamine B to generate red fluorescence (31). All three of these aptamers have potentially improved properties with respect to the Broccoli RNA aptamer.

The assay reported here made use of a 96-well qPCR instrument. However, it is very likely to be amenable to 384-well plates and further conversion to robotic handling and dispensing systems. Nonetheless, it is difficult to exhaustively covary all reaction conditions. For this study, we focused on core reaction components and additives or supplements commonly reported to affect transcription efficiency. Our results build on previous studies through independent investigation of T7 transcription with a new method. Most results supported and confirmed previous studies. Despite the limitations of this method, it can reveal important aspects of transcription in a rapid manner and allow optimization of features like reaction conditions and promoter sequence. It should also translate well to *in vitro* characterization of other polymerases, including viral, bacterial, and possibly eukaryotic. Our laboratory and others have become interested in selectively slowing RNA polymerase II transcription across microsatellite repeat expansions, for example, which could identify molecules with therapeutic potential to treat certain neurological disorders (55, 56, 57). However, for more complex eukaryotic systems with typically low transcription yields, improvements in sensitivity will likely be needed, such as through transcription of tandem aptamer array reporters.

## Experimental procedures

### Purification of T7 RNAP enzyme

BL21 (DE3) cells transformed with a plasmid expressing T7 RNAP (pT7-911) (58) were cultured and T7 RNAP was purified as previously described (59) with several modifications. Briefly, a 0.5-L culture was grown in LB media at 37 °C to an OD_600_ of 0.5 then expression induced with 1 mM IPTG at 37 °C for an additional 4 h. Cells were pelleted and resuspended in Binding Buffer (20 mM Tris-HCl, pH 7.9, 0.5 M NaCl, 0.05% [v/v] Tween-20, 5 mM imidazole, 1 mM phenylmethylsulfonylfluoride). The cell suspension was sonicated and clarified by centrifugation, and the soluble fraction was purified by affinity chromatography using a 3-ml bed volume of HisPur Cobalt-CMA Resin (Thermo Fisher Scientific). After applying the soluble fraction to the column, the resin was washed with 50 bed volumes (150 ml) of Wash Buffer 1 (20 mM Tris-HCl, pH 7.9, 0.5 M NaCl, 0.05% Tween-20, 30 mM imidazole) and 5 bed volumes (15 ml) of Wash Buffer 2 (40 mM Tris-HCl, pH 7.9, 200 mM NaCl, 2 mM DTT, 30 mM imidazole) and eluted with 5 bed volumes of Elution Buffer (40 mM Tris-HCl, pH 7.9, 200 mM NaCl, 2 mM DTT, 200 mM imidazole). The purified protein was concentrated with Vivaspin 15 centrifugal concentrators (Sartorius, 30K MWCO) and the buffer exchanged in the same concentrator using Elution Buffer lacking imidazole to reduce imidazole to 20 mM. One volume of glycerol was added to give a final storage buffer for the T7 RNAP of 20 mM Tris-HCl, pH 7.9, 100 mM NaCl, 1 mM DTT, 10 mM imidazole, and 50% glycerol. The concentration was determined by UV absorbance at 280 nm using a calculated extinction coefficient and Beer’s law. Aliquots of the purified T7 RNAP were flash frozen in liquid nitrogen and stored at −80 °C.

### DNA templates for *in vitro* T7 transcription

Antisense T7-Broccoli DNA templates and the sense T7 promoter DNA oligos were synthesized by Integrated DNA Technologies. Antisense templates and promoter oligos for transcription of sgRNA were also synthesized by Integrated DNA Technologies. For transcription of 7SK-Broccoli RNA, a custom plasmid was generated that expresses human 7SK fused to the Broccoli RNA aptamer behind a Histone H1 promoter. Immediately upstream of the 7SK sequence is also a canonical T7 promoter. The plasmid was linearized with a restriction enzyme directly following the Broccoli aptamer sequence, and run-off transcription was performed. *H. volcanii* tRNA^Trp^ (49) was PCR amplified to include a T7 promoter to allow transcription following amplification. To prepare oligonucleotide templates, complementary T7 promoter DNA oligonucleotides were annealed to the antisense T7 DNA templates at a 1:1 M ratio in 10 mM Tris, pH 7.4, 0.1 mM EDTA. Samples were heated to 95 °C for 5 min then slow cooled to room temperature in a heating block over 1 h. This forms a hybrid single-stranded T7 DNA template where only the promoter sequence is double stranded. Oligonucleotide and RNA sequences are listed in Table S1.

### Quantitative fluorescence readout of transcription using a qPCR instrument

Transcription reactions (10 μl) were assembled in a 96-well qPCR plate on ice. Standard T7 transcription reactions contained 30 mM Tris-HCl, pH 7.9, 30 mM MgCl_2_, 2 mM spermidine, 5 mM NaCl, 1 mM KCl, 10 mM DTT, 50 μg/ml BSA, 1.67% DMSO, 0.005% Triton X-100, 2% polyethylene glycol (PEG_8000_), 5 mM of each ribonucleoside triphosphate (20 mM total rNTPs), 2.5 μM synthetic DNA template, 3.5 μM T7 RNAP, 0.0025 units of inorganic PPase (Thermo Fisher Scientific), and 60 μM DFHBI-1T (Lucerna Technologies). The presence of KCl is to ensure sufficient quadruplex formation of the Broccoli RNA aptamer (29). Under our conditions, the presence of PPase was not necessary for high transcription yield. However, it was required during fluorescence-based measurements to prevent cloudiness due to inorganic pyrophosphate precipitation with magnesium as the reaction proceeds. The presence of DMSO is for solubility of DFHBI-1T, which is dissolved in DMSO solution. To fully dissolve the DFHBI-1T solution prior to reaction assembly, thaw completely then heat temporarily to 65 °C for 5 min and cool to room temperature. For assembly of reactions, 1 μl of 0.3 M MgCl_2_ was first spotted into each well of a 96-well qPCR plate then incubated on ice for 15 min while master stocks were prepared. In general, a 10X Transcription Buffer was prepared that contained 300 mM Tris-HCl, pH 7.9, 20 mM spermidine, 10 mM KCl, 0.05% Triton X-100, and 20% polyethylene glycol (PEG_8000_). Other reagents were added separately to prepare the master stock. Typically, rNTPs were 100 mM commercial stocks at neutral pH (Thermo Fisher Scientific). Particular reagents being tested were omitted and titrated as appropriate. Once the final master stocks were prepared, these were incubated on ice for 10 min then 9 μl was added to respective wells of the 96-well plate. The plate was equilibrated on ice in a 4 °C refrigerator for 15 min prior to moving to a qPCR instrument for reading.

The prepared plate was placed in a Bio-Rad C1000 Thermal Cycler with a CFX96 Real-Time System where the heating block was set to 4 °C. The reaction was initiated by ramping the temperature up to 37 °C, and the fluorescence intensity of each well was collected every 1 min for 60 min using the SYBR filter. Select representative time points, rather than all 60 time points, were chosen for simplicity of data presentation. Reading times for qPCR instruments vary; however, we found that a 53-s incubation time was necessary to allow for the 7-s read time over each 1-min reading interval when reading the entire plate with a single filter (SYBR) in the CFX-96 instrument. Raw fluorescence (no baseline subtraction) was collected for data analysis. Fluorescence at the first time point was subtracted to begin at zero for each sample. Fluorescent readings were normalized to the max fluorescence reading for the standard amount of the reagent. Thus, fluorescence is reported relative to the standard reaction conditions whenever normalized fluorescence data are presented. When specific buffer components or conditions were varied, they were generally normalized to the typical concentration of that component or condition under standard reaction conditions. Curve fitting and equation development were performed in Prism (GraphPad).

### Polyacrylamide gel electrophoresis of transcription products

Transcription samples were typically pooled from three separate fluorescence transcription reactions. For nonfluorescent RNA products, 20-μl reactions were prepared and the entire reaction was used for PAGE analysis. Samples were typically treated with 1 U of DNase I for 15 min at 37 °C, EDTA was added to a final of 60 mM, 1 volume of Loading Buffer was added (90% formamide, 1X TBE, 0.1% SDS), boiled at 95 ˚C for 5 min, then resolved on a denaturing (6 M urea) 15% TBE polyacrylamide gel. The RNA product bands were stained with methylene blue then destained with water. Bands were quantified with ImageJ. Briefly, gel images were saved as .jpeg or .tif files then opened in ImageJ. The gel should be horizontal. The “rectangular” tool was selected and a box was drawn to include the Broccoli RNA bands across all lanes. To quantify the bands, we went to the “Analyze” tab, then clicked on “Gels” in the drop-down menu, then clicked on “Select First Lane.” After confirming first lane selection by clicking “Yes” in the pop-up box, we then followed the same path and clicked “Select Next Lane” and then again clicked on “Plot Lanes.” These last three steps can be accomplished with the shortcut of holding down the control key and pressing the 1, 2, and 3 number keys. Once the lane plot window opened, a line that is approximately horizontal was drawn under the histogram peaks. This line matched with and connected the background levels in the plot. Finally, the peaks are quantified by selecting the “wand (tracing)” tool and clicking in the enclosed area of each peak. The values given in the new “Results” window that opens are taken as the quantification of each peak.

## Data availability

Full data sets, including fluorescence time course data and gel images, may be available upon request to the corresponding author.

## Conflict of interest

The authors declare that they have no conflicts of interest with the contents of this article.
