# Supplementary material for: Revisiting T7 RNA polymerase transcription in vitro with the Broccoli RNA aptamer as a simplified real-time fluorescent reporter
Source: J Biol Chem. 2020 Dec 16;296:100175. doi: 10.1074/jbc.RA120.014553 (PMC7948468; doi:10.1074/jbc.RA120.014553)
Supplement: Supplementary Figures and Table [file mmc1.pdf]

SUPPLEMENTAL INFORMATION

Revisiting T7 RNA polymerase transcription *in vitro* with the Broccoli RNA aptamer as a simplified real-time fluorescent reporter

Zachary J. Kartje[1,†], Helen I. Janis[1,§], Shaoni Mukhopadhyay[2], and Keith T. Gagnon[1,2,*]

[1]Department of Chemistry and Biochemistry, Southern Illinois University, Carbondale, IL, USA.
[2]Department of Biochemistry and Molecular Biology, Southern Illinois University School of Medicine, Carbondale, IL, USA.
[†]Present Address: RNA Therapeutics Institute, University of Massachusetts Medical School, Worcester, MA, USA.
[§]Present Address: Department of Chemistry and Biochemistry, University of Arizona, Tucson, AZ, USA.
[*]Corresponding Author: ktgagnon@siu.edu

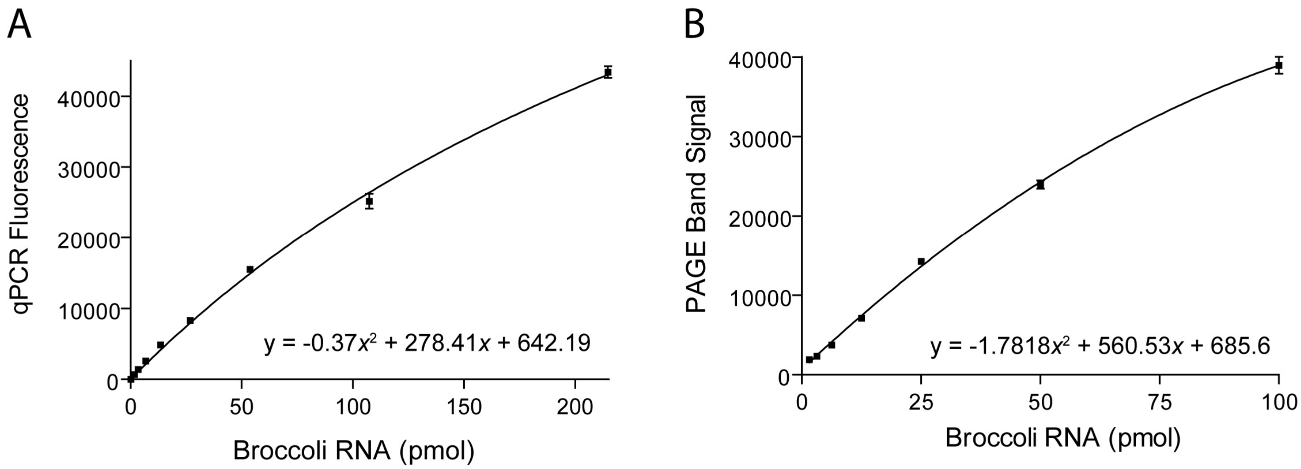

<u>Figure S1</u>. Standard curves for correlating fluorescence intensity or methylene blue staining of Broccoli RNA to molar concentrations. Increasing concentrations of Broccoli RNA gel-purified and quantified by absorbance and 260 nm and using a calculated extinction coefficient were measured for fluorescence (**A**) or band intensity on a methylene blue-stained denaturing polyacrylamide gel quantified by ImageJ analysis (**B**). Non-linear regression in Prism was used to fit equations and solve for pmols of Broccoli RNA starting with fluorescence intensity or band intensity by staining. Two independent replicates were used for PAGE quantification. All error bars are S.E.M.

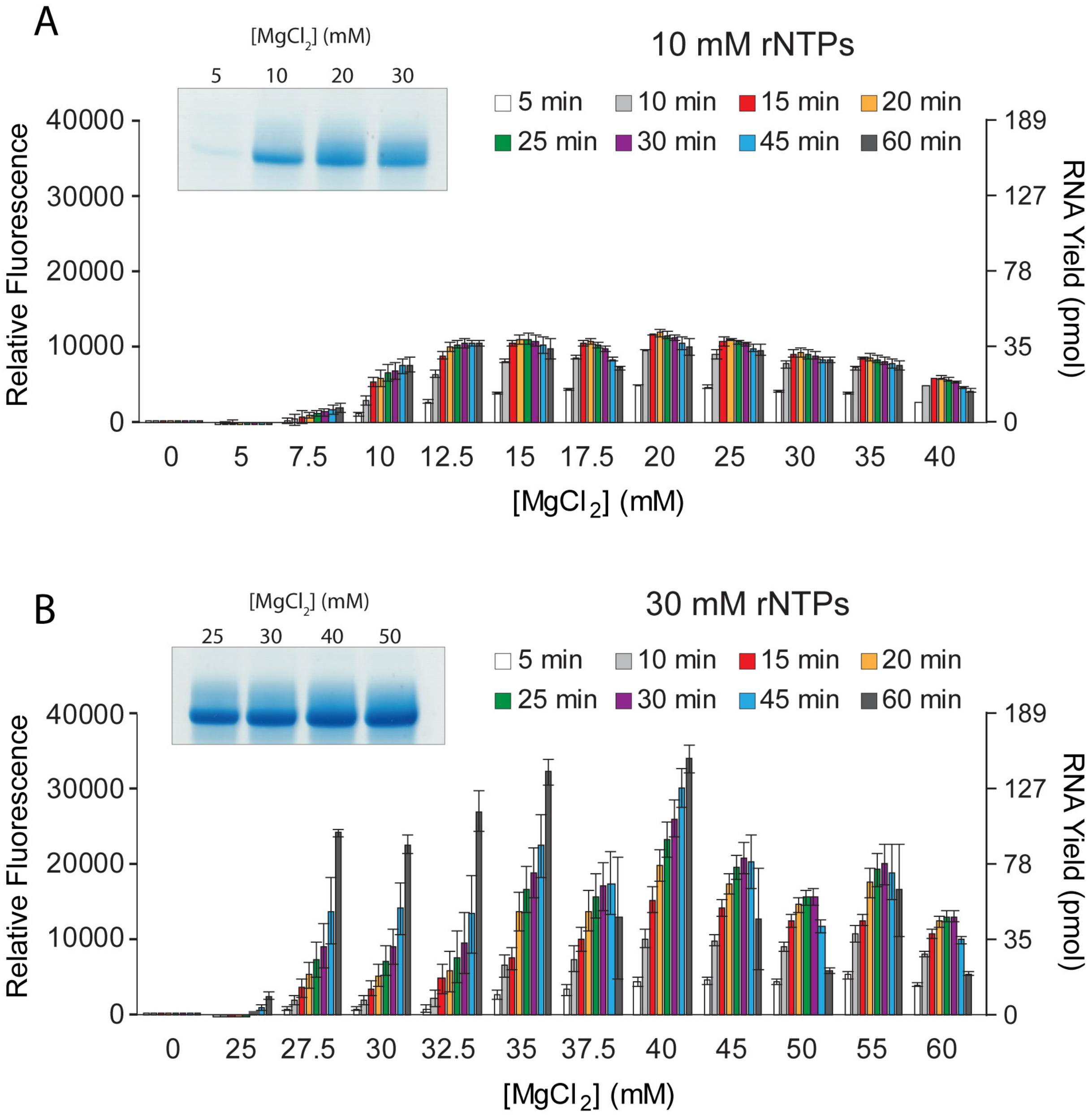

<u>Figure S2</u>. Effect of $MgCl_2$ concentration on *in vitro* T7 transcription efficiency at variable rNTP concentrations. Titration of $MgCl_2$ into reactions containing (**A**) 10 mM total rNTPs or (**B**) 30 mM total rNTPs. Relative fluorescence or RNA yield was measured over time. Insets are three pooled reactions from 60 min time points resolved by denaturing PAGE. Error bars are S.E.M.

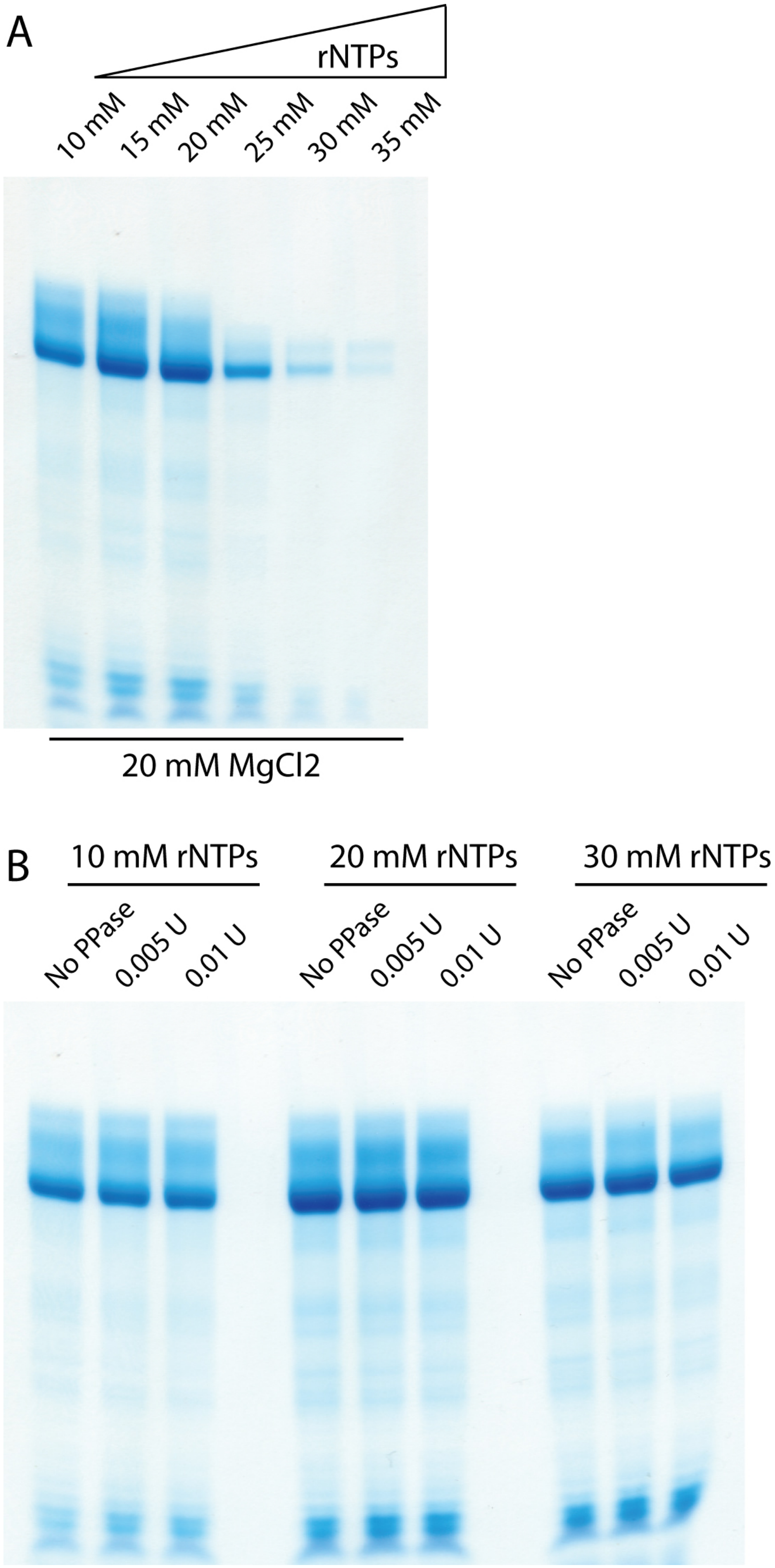

<u>Figure S3</u>. Titration of rNTPs at constant $MgCl_2$ concentration and titration of pyrophosphatase at variable rNTP and MgCl2 concentrations. (**A**) Titration of rNTPs from 10 mM to 35 mM at constant 20 mM $MgCl_2$ in standard *in vitro* T7 transcription reactions after 60 min resolved by denaturing PAGE. (**B**) Titration of pyrophosphatase (PPase) into standard *in vitro* T7 transcription reactions at 10 mM, 20 mM or 30 mM total rNTPs containing 20 mM, 30 mM and 40 mM $MgCl_2$, respectively. PPase was added at 5-fold or 10-fold the usual concentration of standard fluorescent reactions. All reactions were stopped at 60 min, treated with 1 unit of DNase I for 15 min, and EDTA added to 60 mM (to dissolve magnesium-pyrophosphate precipitates), then resolved by denaturing PAGE. Precipitation was heavy in No PPase reactions but not observable in PPase-treated samples.

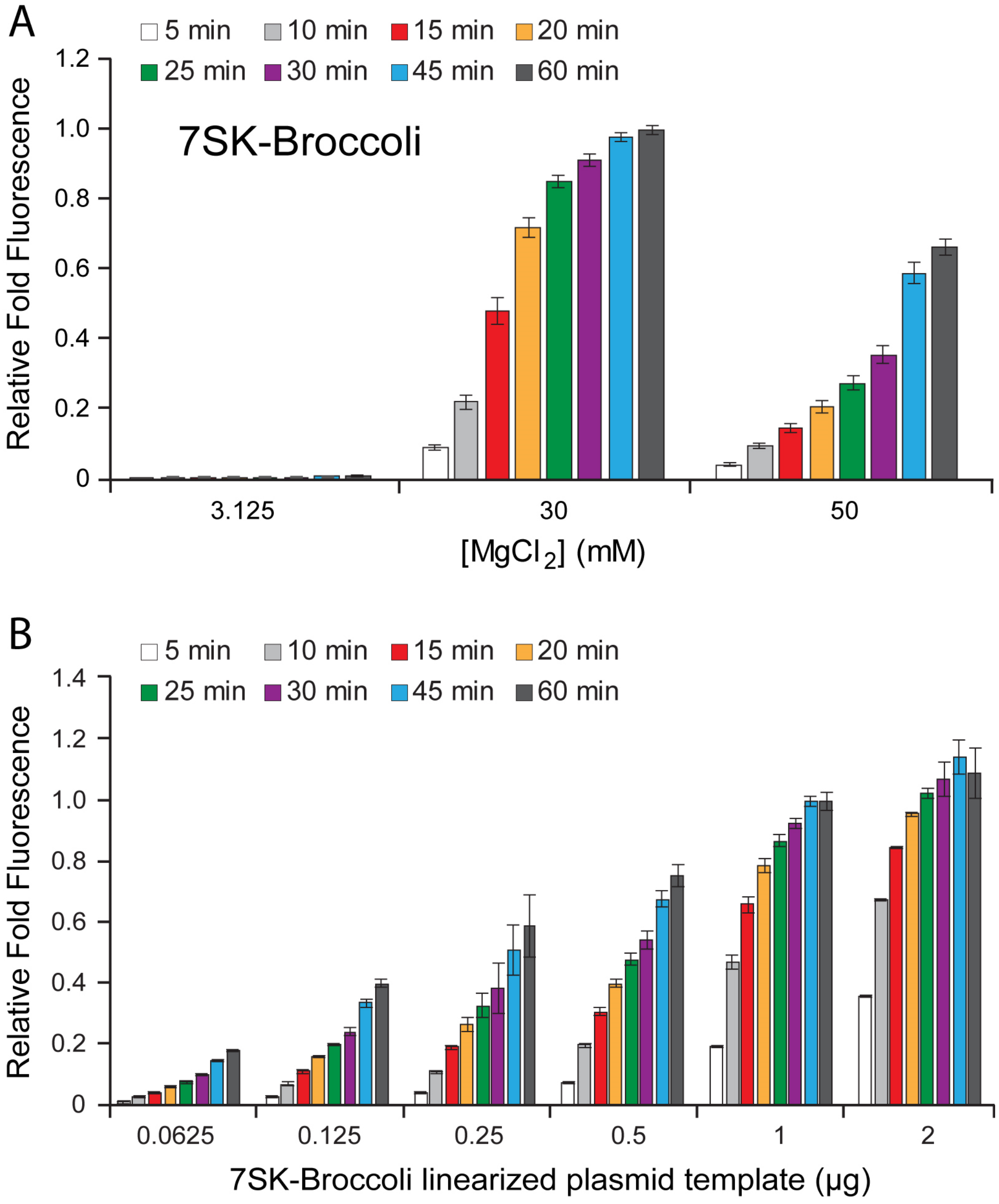

Figure S4. Effect of $MgCl_2$ concentrations and plasmid DNA template concentrations on *in vitro* T7 transcription efficiency of human 7SK-Broccoli RNA. (**A**) Three variable $MgCl_2$ concentrations were tested for efficiency of 7SK-Broccoli RNA transcription efficiency from a linearized plasmid template. (**B**) Titration of linearized 7SK-Broccoli plasmid template into standard transcription reactions. For all panels, relative fold fluorescence from Broccoli RNA aptamer was measured over time and normalized to standard transcription efficiencies. Error bars are S.E.M.

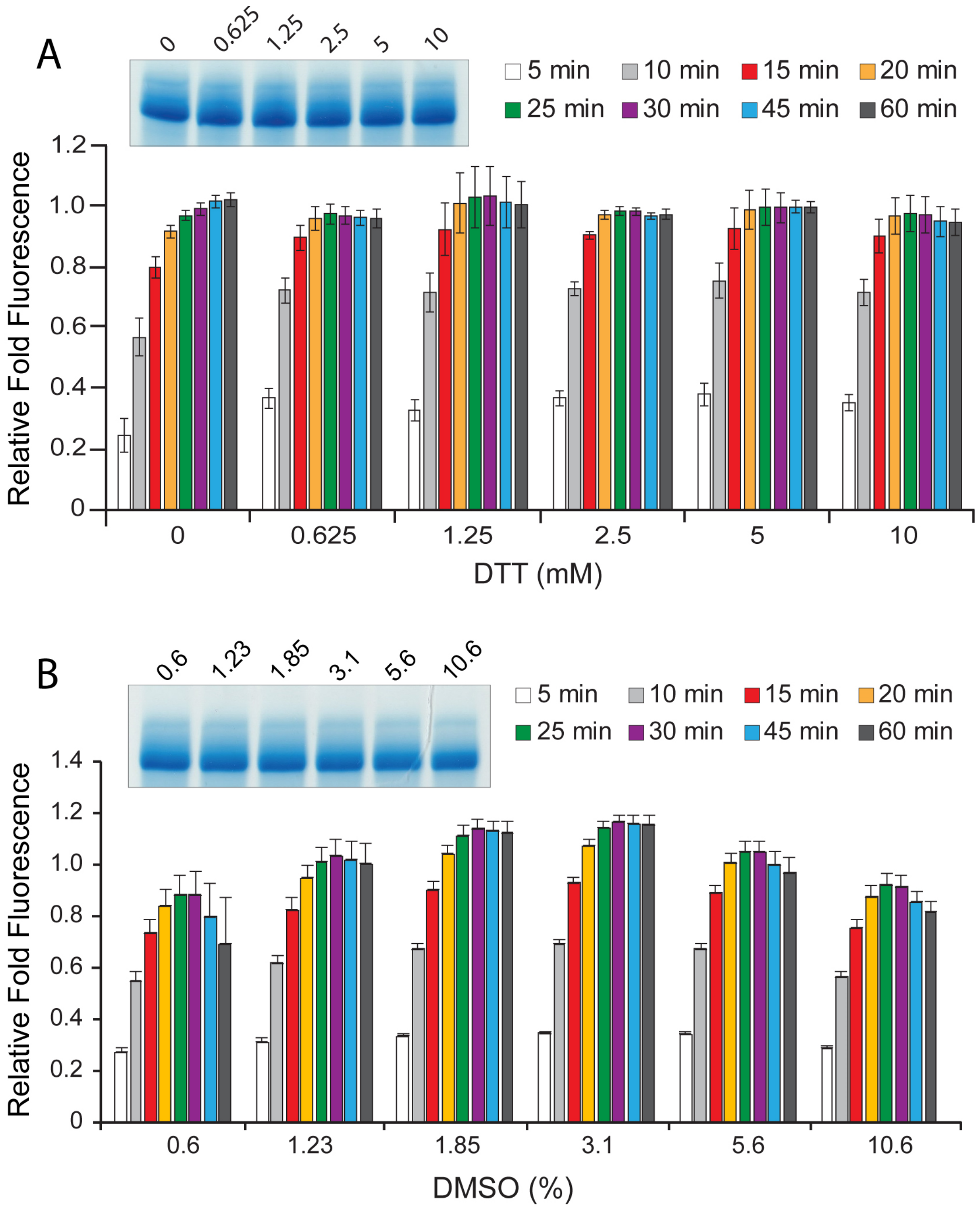

<u>Figure S5</u>. Effect of DTT and DMSO concentrations on *in vitro* T7 transcription efficiency. (**A**) Increasing concentrations of DTT in standard fluorescent T7 transcription reactions. (**B**) Increasing concentrations of DMSO in standard fluorescent T7 transcription reactions. For all panels, relative fold fluorescence is normalized to standard conditions. Insets are three pooled reactions from 60 min time points resolved by denaturing PAGE. Error bars are S.E.M.

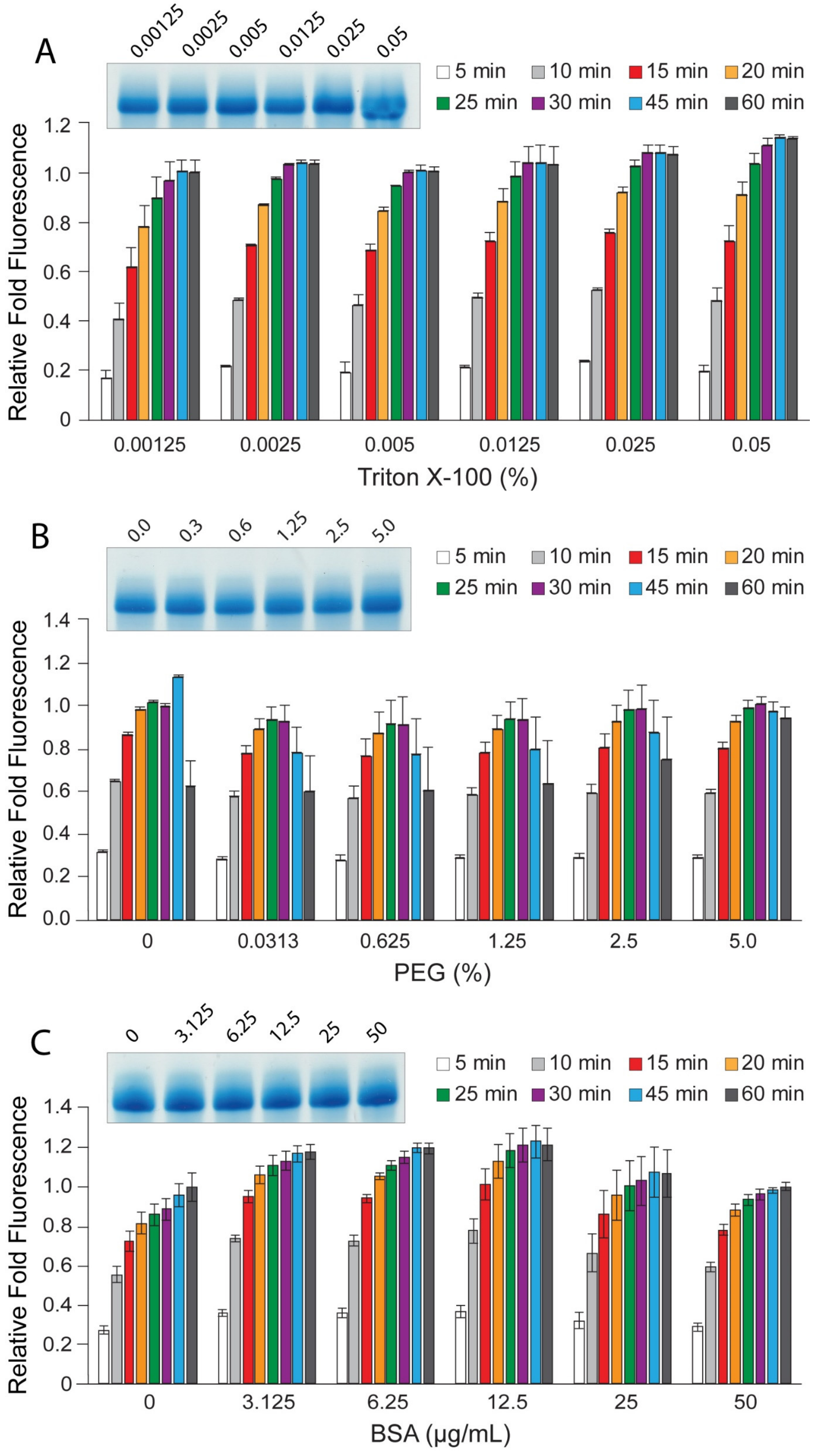

<u>Figure S6</u>. Effect of Triton X-100, PEG and BSA on *in vitro* T7 transcription efficiency. Increasing concentrations of Triton X-100 (**A**), PEG_{8000} (**B**), and BSA (**C**) in standard fluorescent T7 transcription reactions. For all panels, relative fold fluorescence is normalized to standard conditions. Insets are three pooled reactions from 60 min time points resolved by denaturing PAGE. Error bars are S.E.M.

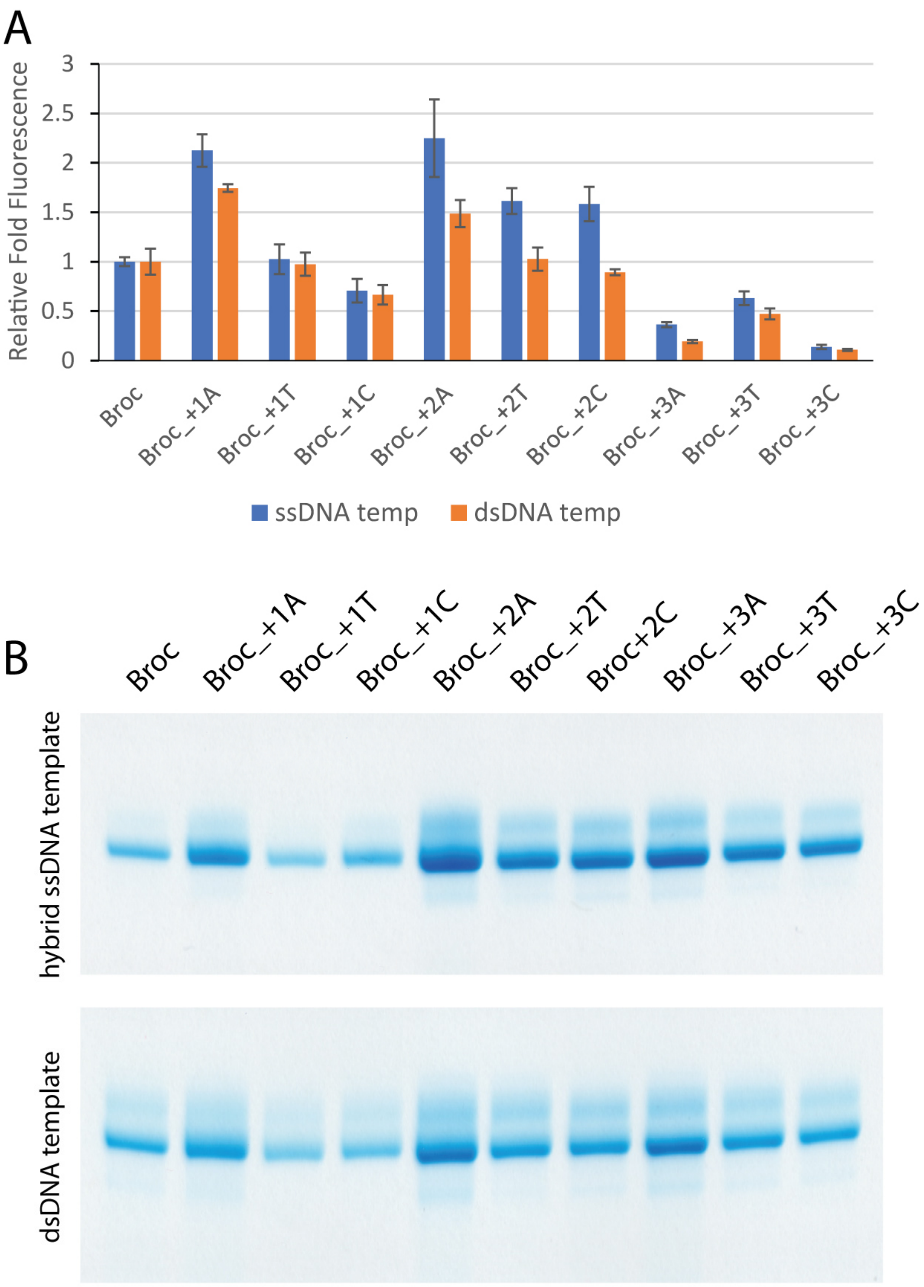

<u>**Figure S7**</u>**. *In vitro* T7 transcription using ssDNA hybrid templates versus fully dsDNA templates for the Broccoli RNA aptamer.** (**A**) Comparing relative fold fluorescence of ssDNA hybrid and dsDNA templates for mutants at positions +1, +2, and +3 of the T7 promoter. Error bars are S.E.M. (**B**) Denaturing PAGE of three pooled fluorescent reactions (shown in panel A) at 60 min time points for mutants at positions +1, +2, and +3. The upper panel is the same as shown in Figure 6A inset so as to provide a direct visual comparison of RNA products from ssDNA hybrid versus dsDNA templates.

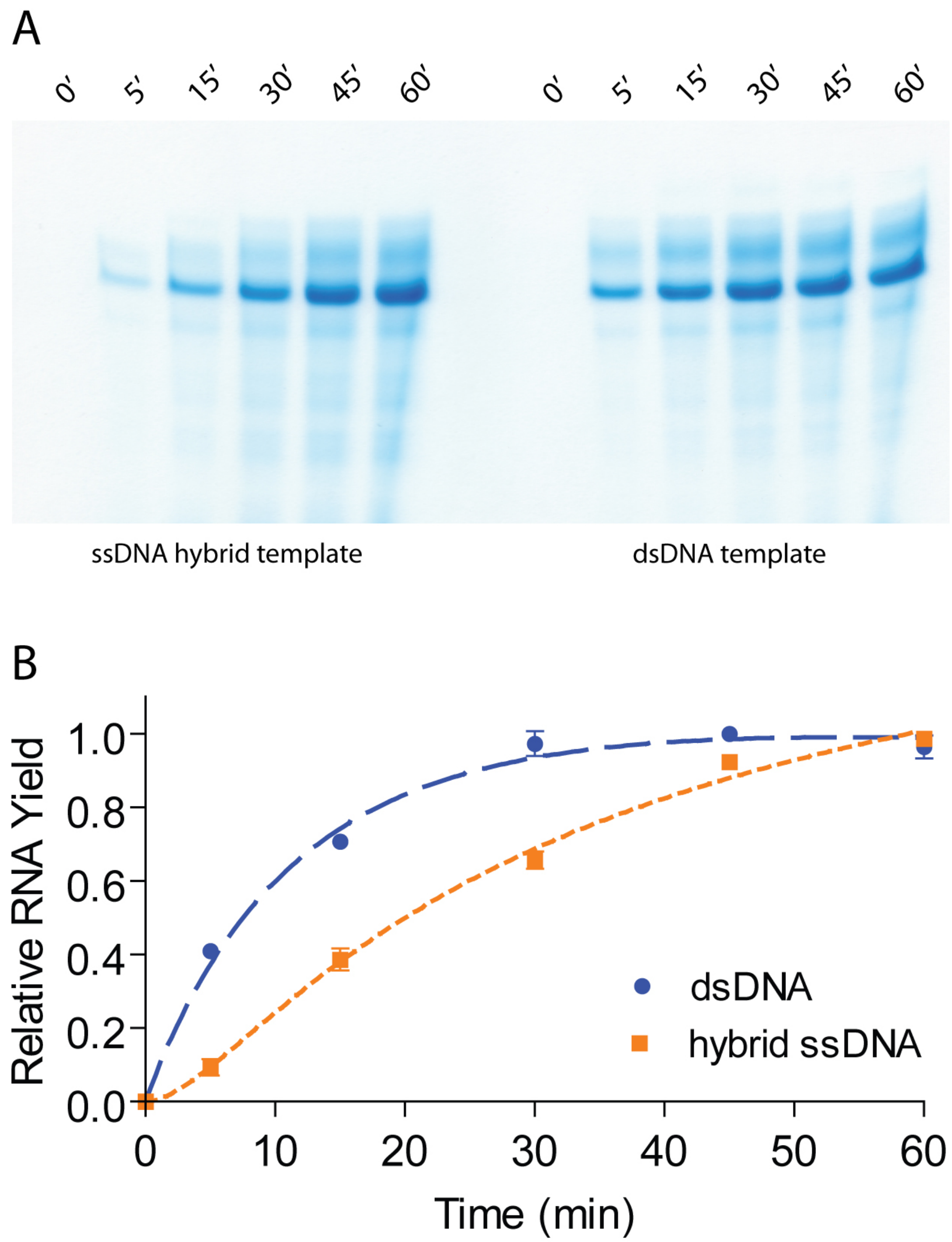

<u>Figure S8</u>. **Time course of *in vitro* T7 transcription for the Broccoli RNA aptamer quantified by gel electrophoresis.** (**A**) Representative denaturing PAGE of *in vitro* T7 transcription time course reactions for ssDNA hybrid and dsDNA templates. Time points were taken at 0, 5, 15, 30, 45, and 60 min. (**B**) Quantification of two replicate denaturing PAGE gels by methylene blue staining. Relative RNA yield demonstrated that dsDNA templates facilitated faster reaction kinetics but relative yield was the same for both templates by 60 min. Error bars are S.E.M.

Table S1: Oligonucleotides and RNAs

Name	Sequence
T7 promoter DNA oligo	TAATACGACTCACTATAG
anti-T7 broccoli	GAGCCCACACTCTACTCGACAGATACGAATATCTGGACCCGACCGTCTCccctatagtgagtcgtatta
sense-T7 broccoli	taatacgactcactatagggGAGACGGTCGGGTCCAGATATTCGTATCTGTCGAGTAGAGTGTGGGCTC
anti-T7 sgRNA	AAGCACCGACTCGGTGCCACTTTTTCAAGTTTGATAACGGACTAGCCTTATTTTAACTTGCTATTTCTAGCTCTAAAACATCGGAGTGTTTTGCTGGTAccctatagtgagtcgtatta
Hv $tRNA^{Trp}$	GGGGGCUGUGGCCAAGCCCGGCAUGGCGACUGACUCCAGAUCAGUCGAUCGGGGGUUCAAAUCCCUCCGGCCCCA
T7-broc_1A	GAGCCCACACTCTACTCGACAGATACGAATATCTGGACCCGACCGTCTCccctatagtgagtcgtattT
T7-broc_1C	GAGCCCACACTCTACTCGACAGATACGAATATCTGGACCCGACCGTCTCccctatagtgagtcgtattG
T7-broc_1G	GAGCCCACACTCTACTCGACAGATACGAATATCTGGACCCGACCGTCTCccctatagtgagtcgtattC
T7-broc_2T	GAGCCCACACTCTACTCGACAGATACGAATATCTGGACCCGACCGTCTCccctatagtgagtcgtatAa
T7-broc_2C	GAGCCCACACTCTACTCGACAGATACGAATATCTGGACCCGACCGTCTCccctatagtgagtcgtatGa
T7-broc_2G	GAGCCCACACTCTACTCGACAGATACGAATATCTGGACCCGACCGTCTCccctatagtgagtcgtatCa
T7-broc_3T	GAGCCCACACTCTACTCGACAGATACGAATATCTGGACCCGACCGTCTCccctatagtgagtcgtaAta
T7-broc_3C	GAGCCCACACTCTACTCGACAGATACGAATATCTGGACCCGACCGTCTCccctatagtgagtcgtaGta
T7-broc_3G	GAGCCCACACTCTACTCGACAGATACGAATATCTGGACCCGACCGTCTCccctatagtgagtcgtaCta
T7-broc_4A	GAGCCCACACTCTACTCGACAGATACGAATATCTGGACCCGACCGTCTCccctatagtgagtcgtTtta
T7-broc_4C	GAGCCCACACTCTACTCGACAGATACGAATATCTGGACCCGACCGTCTCccctatagtgagtcgtGtta
T7-broc_4G	GAGCCCACACTCTACTCGACAGATACGAATATCTGGACCCGACCGTCTCccctatagtgagtcgtCtta
T7-broc_5T	GAGCCCACACTCTACTCGACAGATACGAATATCTGGACCCGACCGTCTCccctatagtgagtcgAatta
T7-broc_5C	GAGCCCACACTCTACTCGACAGATACGAATATCTGGACCCGACCGTCTCccctatagtgagtcgGatta
T7-broc_5G	GAGCCCACACTCTACTCGACAGATACGAATATCTGGACCCGACCGTCTCccctatagtgagtcgCatta
T7-broc_6A	GAGCCCACACTCTACTCGACAGATACGAATATCTGGACCCGACCGTCTCccctatagtgagtcTtatta
T7-broc_6T	GAGCCCACACTCTACTCGACAGATACGAATATCTGGACCCGACCGTCTCccctatagtgagtcAtatta
T7-broc_6G	GAGCCCACACTCTACTCGACAGATACGAATATCTGGACCCGACCGTCTCccctatagtgagtcCtatta
T7-broc_7A	GAGCCCACACTCTACTCGACAGATACGAATATCTGGACCCGACCGTCTCccctatagtgagtTgtatta
T7-broc_7T	GAGCCCACACTCTACTCGACAGATACGAATATCTGGACCCGACCGTCTCccctatagtgagtAgtatta
T7-broc_7C	GAGCCCACACTCTACTCGACAGATACGAATATCTGGACCCGACCGTCTCccctatagtgagtGgtatta
T7-broc_8T	GAGCCCACACTCTACTCGACAGATACGAATATCTGGACCCGACCGTCTCccctatagtgagAcgtatta
T7-broc_8C	GAGCCCACACTCTACTCGACAGATACGAATATCTGGACCCGACCGTCTCccctatagtgagGcgtatta
T7-broc_8G	GAGCCCACACTCTACTCGACAGATACGAATATCTGGACCCGACCGTCTCccctatagtgagCcgtatta
T7-broc_9A	GAGCCCACACTCTACTCGACAGATACGAATATCTGGACCCGACCGTCTCccctatagtgaTtcgtatta
T7-broc_9T	GAGCCCACACTCTACTCGACAGATACGAATATCTGGACCCGACCGTCTCccctatagtgaAtcgtatta
T7-broc_9G	GAGCCCACACTCTACTCGACAGATACGAATATCTGGACCCGACCGTCTCccctatagtgaCtcgtatta
T7-broc_10A	GAGCCCACACTCTACTCGACAGATACGAATATCTGGACCCGACCGTCTCccctatagtgTgtcgtatta
T7-broc_10C	GAGCCCACACTCTACTCGACAGATACGAATATCTGGACCCGACCGTCTCccctatagtgGgtcgtatta
T7-broc_10G	GAGCCCACACTCTACTCGACAGATACGAATATCTGGACCCGACCGTCTCccctatagtgCgtcgtatta
T7-broc_11A	GAGCCCACACTCTACTCGACAGATACGAATATCTGGACCCGACCGTCTCccctatagtTagtcgtatta
T7-broc_11T	GAGCCCACACTCTACTCGACAGATACGAATATCTGGACCCGACCGTCTCccctatagtAagtcgtatta
T7-broc_11G	GAGCCCACACTCTACTCGACAGATACGAATATCTGGACCCGACCGTCTCccctatagtCagtcgtatta
T7-broc_12T	GAGCCCACACTCTACTCGACAGATACGAATATCTGGACCCGACCGTCTCccctatagAgagtcgtatta
T7-broc_12C	GAGCCCACACTCTACTCGACAGATACGAATATCTGGACCCGACCGTCTCccctatagGgagtcgtatta
T7-broc_12G	GAGCCCACACTCTACTCGACAGATACGAATATCTGGACCCGACCGTCTCccctatagCgagtcgtatta
T7-broc_13A	GAGCCCACACTCTACTCGACAGATACGAATATCTGGACCCGACCGTCTCccctataTtgagtcgtatta
T7-broc_13T	GAGCCCACACTCTACTCGACAGATACGAATATCTGGACCCGACCGTCTCccctataAtgagtcgtatta
T7-broc_13G	GAGCCCACACTCTACTCGACAGATACGAATATCTGGACCCGACCGTCTCccctataCtgagtcgtatta
T7-broc_14A	GAGCCCACACTCTACTCGACAGATACGAATATCTGGACCCGACCGTCTCccctatTgtgagtcgtatta
T7-broc_14C	GAGCCCACACTCTACTCGACAGATACGAATATCTGGACCCGACCGTCTCccctatGgtgagtcgtatta
T7-broc_14G	GAGCCCACACTCTACTCGACAGATACGAATATCTGGACCCGACCGTCTCccctatCgtgagtcgtatta
T7-broc_15T	GAGCCCACACTCTACTCGACAGATACGAATATCTGGACCCGACCGTCTCccctaAagtgagtcgtatta
T7-broc_15C	GAGCCCACACTCTACTCGACAGATACGAATATCTGGACCCGACCGTCTCccctaGagtgagtcgtatta
T7-broc_15G	GAGCCCACACTCTACTCGACAGATACGAATATCTGGACCCGACCGTCTCccctaCagtgagtcgtatta
T7-broc_16A	GAGCCCACACTCTACTCGACAGATACGAATATCTGGACCCGACCGTCTCccctTtagtgagtcgtatta
T7-broc_16C	GAGCCCACACTCTACTCGACAGATACGAATATCTGGACCCGACCGTCTCccctGtagtgagtcgtatta
T7-broc_16G	GAGCCCACACTCTACTCGACAGATACGAATATCTGGACCCGACCGTCTCccctCtagtgagtcgtatta
T7-broc_17T	GAGCCCACACTCTACTCGACAGATACGAATATCTGGACCCGACCGTCTCcccAatagtgagtcgtatta
T7-broc_17C	GAGCCCACACTCTACTCGACAGATACGAATATCTGGACCCGACCGTCTCcccGatagtgagtcgtatta
T7-broc_17G	GAGCCCACACTCTACTCGACAGATACGAATATCTGGACCCGACCGTCTCcccCatagtgagtcgtatta
T7-broc_18A	GAGCCCACACTCTACTCGACAGATACGAATATCTGGACCCGACCGTCTCccTtatagtgagtcgtatta
T7-broc_18T	GAGCCCACACTCTACTCGACAGATACGAATATCTGGACCCGACCGTCTCccAtatagtgagtcgtatta
T7-broc_18C	GAGCCCACACTCTACTCGACAGATACGAATATCTGGACCCGACCGTCTCccGtatagtgagtcgtatta
T7-broc_19A	GAGCCCACACTCTACTCGACAGATACGAATATCTGGACCCGACCGTCTCcTctatagtgagtcgtatta
T7-broc_19T	GAGCCCACACTCTACTCGACAGATACGAATATCTGGACCCGACCGTCTCcActatagtgagtcgtatta
T7-broc_19C	GAGCCCACACTCTACTCGACAGATACGAATATCTGGACCCGACCGTCTCcGctatagtgagtcgtatta
T7-broc_20A	GAGCCCACACTCTACTCGACAGATACGAATATCTGGACCCGACCGTCTCTcctatagtgagtcgtatta
T7-broc_20T	GAGCCCACACTCTACTCGACAGATACGAATATCTGGACCCGACCGTCTCAcctatagtgagtcgtatta
T7-broc_20C	GAGCCCACACTCTACTCGACAGATACGAATATCTGGACCCGACCGTCTCGcctatagtgagtcgtatta
T7-broc_18A_s	taatacgactcactataAggGAGACGGTCGGGTCCAGATATTCGTATCTGTCGAGTAGAGTGTGGGCTC
T7-broc_18T_s	taatacgactcactataTggGAGACGGTCGGGTCCAGATATTCGTATCTGTCGAGTAGAGTGTGGGCTC
T7-broc_18C_s	taatacgactcactataCggGAGACGGTCGGGTCCAGATATTCGTATCTGTCGAGTAGAGTGTGGGCTC
T7-broc_19A_s	taatacgactcactatagAgGAGACGGTCGGGTCCAGATATTCGTATCTGTCGAGTAGAGTGTGGGCTC
T7-broc_19T_s	taatacgactcactatagTgGAGACGGTCGGGTCCAGATATTCGTATCTGTCGAGTAGAGTGTGGGCTC
T7-broc_19C_s	taatacgactcactatagCgGAGACGGTCGGGTCCAGATATTCGTATCTGTCGAGTAGAGTGTGGGCTC
T7-broc_20A_s	taatacgactcactataggAGAGACGGTCGGGTCCAGATATTCGTATCTGTCGAGTAGAGTGTGGGCTC
T7-broc_20T_s	taatacgactcactataggTGAGACGGTCGGGTCCAGATATTCGTATCTGTCGAGTAGAGTGTGGGCTC
T7-broc_20C_s	taatacgactcactataggCGAGACGGTCGGGTCCAGATATTCGTATCTGTCGAGTAGAGTGTGGGCTC
